# Higher levels of GluN1 splice cassettes, C2 and C2’, in hippocampus of aged mice were associated with poor spatial reference memory

**DOI:** 10.1016/j.brainresbull.2025.111502

**Published:** 2025-08-05

**Authors:** Kathy R. Magnusson, Daniel R. Zamzow

**Affiliations:** Linus Pauling Institute and Department of Biomedical Sciences, Carlson College of Veterinary Medicine, Oregon State University, Corvallis, OR 97331, United States

**Keywords:** Aging, Morris water maze, Learning, Memory, NMDA receptor, GluN1, Splice variants

## Abstract

Cognitive decline during aging has been linked to changes in the *N*-methyl-D-aspartate receptor (NMDAR). Age-related changes in the GluN1 splice cassette proteins have been described in crude synaptosomes, but synaptic and extrasynaptic NMDARs have different impacts on synaptic plasticity. The present study examined the association between cognitive function and C-terminal splice cassette proteins, C1, C2, and C2’, in different compartments of the synaptic environment. Young and old C57BL/6 male mice were tested for reference memory and cognitive flexibility in the Morris water maze. The older mice were separated into good and poor reference memory groups based on performance during the acquisition phase. The old mice with poor memory acquisition showed increased levels of the C2 protein in the synaptic membrane and the C2’ protein in the extrasynaptic membranes in the hippocampus as compared to old mice with good memory or young, respectively. In the frontal cortex, C2 and C2’ protein levels in the extrasynaptic membrane were associated with good cognitive flexibility across ages. Thus, although alternative splice forms of the GluN1 subunit appear to contribute to cognitive declines during aging, the complexity of these changes and relationships suggest that interventions involving manipulating splicing of the C-terminal tail of the GluN1 subunit would likely exacerbate memory or cognitive flexibility problems or both in aged individuals.

## 1. Introduction

By 2050, the cohort aged ≥ 65 years is projected to increase to 23 % of the U.S. population [109]. Aging-related deficits in brain functions impact memory, cognitive flexibility, strength, sensation, balance and motor coordination, and affect almost half of humans aged ≥ 65 years [105,1]. Memory is the earliest-impaired cognitive function, as people in their late 40’s already show significant deficits in delayed information recall as compared to young adults [1]. In addition, the dementia of Alzheimer’s Disease (AD) develops on top of such aging-related declines [54]. Understanding the underlying causes of age-related cognitive decline could lead to interventions to improve healthspan and delay the onset of debilitation in AD.

Glutamate is the main excitatory neurotransmitter in mammalian brains [27,28]. Glutamate receptors, such as N-methyl-D-aspartate receptors (NMDARs), are present in high density in the brain regions most affected by AD (hippocampus and cortex) [59,62] and play important roles in synaptic transmission, memory, and cognitive flexibility in these regions [19,86,79,73,10,15,66,70,94]. NMDAR ligand-binding densities in the frontal cortex and hippocampus are correlated with spatial reference (long-term) memory [59,61]. Alterations in expression and palmitoylation of NMDARs in the frontal cortices of mice also appear to be involved in cognitive flexibility [121,122].

Aging animals exhibit declines in NMDAR binding densities [9,49,58,88,91,102,112], NMDA-stimulated transmitter release [34,93], induction and maintenance of NMDAR-dependent long-term potentiation [4,5,13,22,21,29], and spatial reference memory attributed to NMDARs [59,61,91]. We have found that NMDARs are more vulnerable to aging than other glutamate receptors [62]. Declines in NMDAR densities documented across aging in the frontal cortex [59,61] and hippocampus [59,61,91] correlate with poor spatial reference memory and blockade of NMDARs mimics age-related deficits in expansion of hippocampal place fields [25,101], suggesting that diminished NMDAR functionality contributes to aging-related spatial memory deficits.

NMDARs are composed of 4 protein subunits [30], which include members of the GluN1, GluN2, and/or GluN3 families [42,44,50,72,77,78,100,114,26,69]. Synaptic NMDARs stimulate synaptic plasticity and promote cell survival, via anti-apoptotic and anti-oxidant mechanisms [35]. Extrasynaptic NMDARs are involved in excitotoxicity, via activation of pro-cell death genes [35,119]; however, there is also evidence that extrasynaptic NMDARs can regulate both long term-potentiation and -depression [55,56,116].

**Fig. 1.**
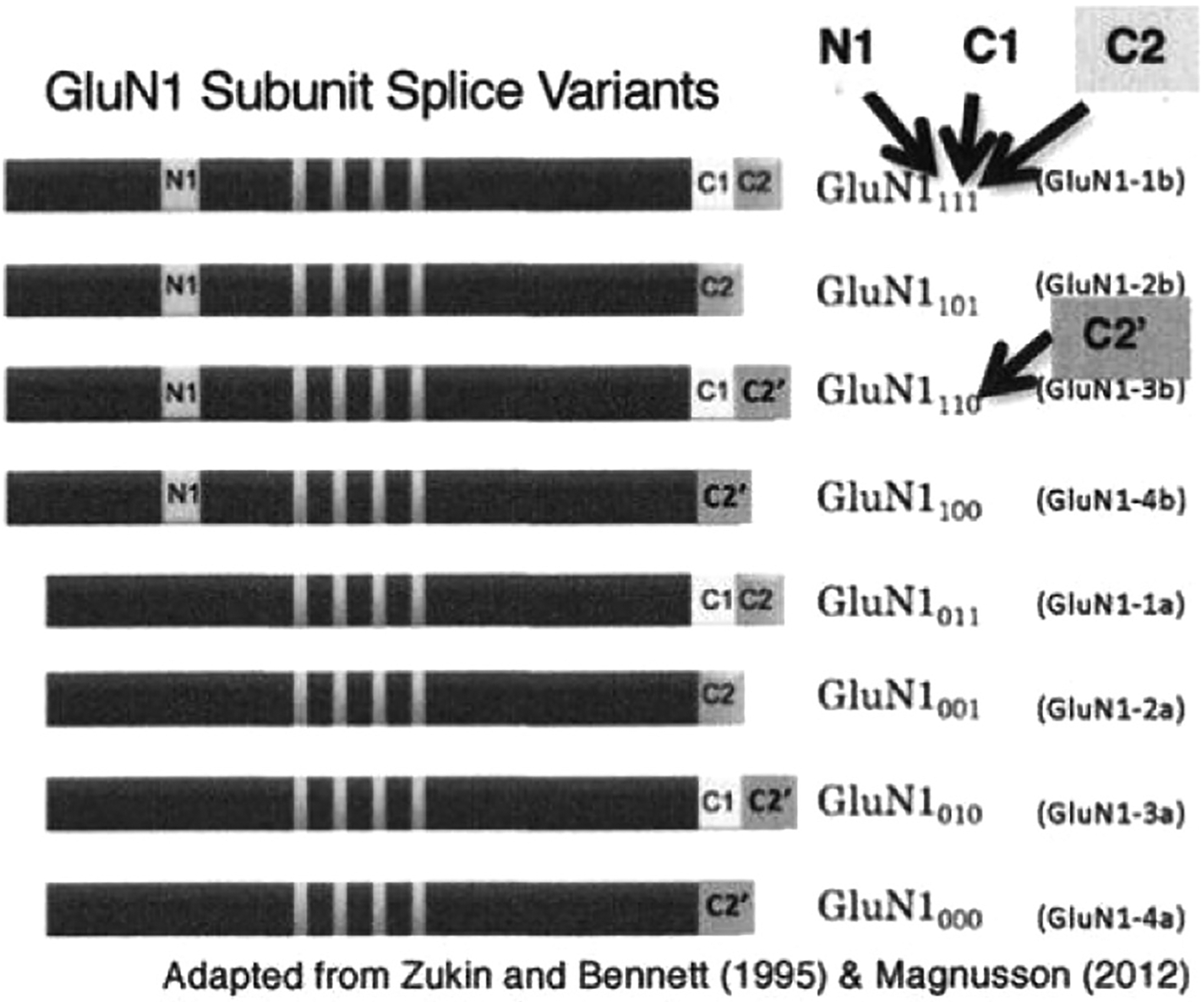
GluN1 splice variants showing the position of cassettes (N1, C1, and C2 cassettes and alternative sequence C2’) and the abbreviations used to identify them.

The GluN1 subunit appears to be necessary and sufficient for the formation of functional NMDAR channels [44,50,72,77]. Eight different splice variants of mRNA exist for the GluN1 subunit through alternative splicing of one N-terminal and two C-terminal cassettes, C1 and alternatively C2 or C2’ [52,126] (Fig. 1). The mRNA for C-terminal splice forms, GluN1–1 (+ C1 and + C2 cassettes) and GluN1–3 (+ C1 and + C2′), show significant declines during aging in frontal cortex and/or hippocampal subregions [16,63]. In crude synaptosomes from prefrontal/frontal cortical regions, C2 and C2’ cassette proteins show declines with increasing age and the GluN1 subunit as a whole and C1 and C2’ cassettes show relationships to spatial reference memory [17].

There is evidence that the remaining NMDARs in aged animals may play a contrasting role to those in young, becoming detrimental to memory and other functions [106,87,123], as poorer learners among aged rodents show higher hippocampal NMDAR binding and GluN1 and GluN2B protein levels than the old good learners [106,123], and an NMDAR antagonist improved neurogenesis [87]. In the frontal cortex of aged mice, we found alterations in GluN2B in the synaptic environment; an increased association of GluN2B with GluN2A and scaffolding proteins and decreased phosphorylation and trafficking in the poorer old learners [121,120]. In crude synaptosomes from frontal cortex, those aged animals with higher levels of GluN1 and C1 and C2’ proteins were associated with poorer performance in a spatial reference memory task [17] or the relationship of these proteins and memory changed between middle-age and older age. It is not clear whether these aging changes that appear to be detrimental to cognitive function are restricted to synaptic NMDARs or also involve extrasynaptic NMDARs.

In the present study we addressed the hypothesis that age-related changes in C-terminal splice variants within different compartments of the synaptic environment would vary by learning and memory ability. Mice were tested for spatial reference memory and cognitive flexibility in the Morris water maze. We utilized Gallagher’s method [32] to separate aged mice into good and poor reference memory groups (old good RM and old poor RM, respectively) and compared them to each other and young mice. Following behavioral testing, the frontal cortex, including prefrontal regions, and hippocampus were separately subfractionated. These same mice were used to assess GluN2B and GluN2A subunits of the NMDAR and associated proteins, in a previous publication [121]. We found significant alterations in the C2 and C2’ cassettes in the synaptic and/or extrasynaptic membranes from the hippocampus of old mice with poor reference memory and correlations with reversal trials.

## 2. Materials and methods

### 2.1. Animals

Twenty-four male C57BL/6 mice from two different age groups (3 & 24 months) were used for the experiment. The twelve older mice were acquired from the National Institute on Aging’s rodent colony at National Institutes of Health (NIH) and the 12 young mice were procured from JAX mice (Bar Harbor, MA), which is the source of the NIH colony. Mice were fed a standard chow diet (LabDiet) *ad libitum* and housed under a 12/12 h light/dark cycle. Within 6 h after completion of behavioral testing, all mice were euthanized by exposure to CO_2,_ followed by decapitation. Brains were removed, frozen by burying in dry ice, and stored in a −80°C freezer.

### 2.2. Behavioral testing

The Morris water maze was used to assess spatial reference memory, cognitive flexibility and associative memory (cued control task) using previously described protocols [18]. Briefly, mice were: 1) acclimated to the water maze for 2 days, 2) assessed for spatial reference memory testing for 2 days, 3) tested for cognitive flexibility with reversal training for 1 day and 4) examined for associative memory in a cued control task for 1 day. Spatial reference memory testing on day 1 consisted of 1) a naïve probe trial, 2) 4 place trials, 3) 1 h cage rest, 4) 4 place trials, 5) 1 h rest, and 6) probe trial. Day 2 was similar, except there was no initial probe trial. The platform remained in the same location during the place trials. Place trials allowed for a maximum of 60 s of searching for the platform, 30 s rest on the platform and at least 2 min of cage rest between place trials. If the animal failed to find the platform within the maximum 60 s trial time, the experimenter led it to the platform. Probe trials were designed to assess the animal’s ability to show a bias for the platform location. The platform was removed and the animal searched for 30 s. Cognitive flexibility was assessed with a reversal task. The platform was moved to the opposite quadrant and testing was conducted similar to Day 2 of reference memory testing. The control trials were utilized to assess physical ability, motivation, and visual acuity in the water maze. Mice underwent 6 cued trials, with the entry points and platform positions altered between trials. The platform was submerged, but was identified by a flag on a 20.3 cm stick. Mice searched for a maximum of 60 s. The SMART tracking system (San Diego Instruments, San Diego, CA) was used to track the animal’s path and proximity to the platform location.

### 2.3. Subfractionation of neural tissue

The frontal cortex, consisting of the rostral 4 mm of cortex, and the hippocampus were dissected and biochemically fractionated as previously described [24], with some modifications. In brief, brain tissue was homogenized in a chilled Dounce homogenizer with the use of ice-cold TE buffer (Tris–HCl (10 mM), pH 7.4; EDTA (1 mM), EGTA (1 mM)) plus sucrose (320 mM) and protease inhibitor cocktail (Sigma). Homogenate was centrifuged for 10 min at 1000 × g and 4°C in an Eppendorf centrifuge. The pellet (P1) obtained was discarded. The supernatant (S1) was centrifuged for 20 min at 12000 × g and 4°C, which resulted in a crude synaptosome pellet (P2) and supernatant consisting of the cytosolic and microsomal fraction (S2). The P2 fraction was solubilized with Triton X-100 (Sigma) and fractionated according to Milnerwood and colleagues [74]. In brief, the P2 pellet was resuspended with 300 μl of Triton buffer (Tris-HCl (10 mM), NaCl (100 mM), Triton (0.5 %), pH 7.2), slowly rotated for 15 min at 4°C, and centrifuged at 12,000 *g* and 4°C for 20 min. The triton-soluble fraction, (TxS; containing non-PSD membranes) was saved as supernatant. The pellet was suspended in 150 μl of SDS buffer (Tris-HCl (10 mM), NaCl (150 mM), Triton-X (1 %), deoxycholic acid (1 %), SDS (1 %), DTT (1 mM), pH 7.5), gently rotated at 4°C for 1 h and centrifuged at 10,000 *g* and 4°C for 15 min. The triton insoluble PSD fraction (TxP) in the supernatant was saved. PSD (TxP), non-PSD (TxS), and microsomal and cytosolic (S2) fractions were stored at −80°C until assayed.

### 2.4. Semi-quantiative Western blot

Semi-quantitative Western blotting was conducted with the use of sodium dodecyl sulfate–polyacrylamide gel (10 %) electrophoresis as previously published [121,123]. Four different μg loads (2, 4, 8 and 16 μg/well) of standards (homogenate from combined caudal cortices of all young animals) were present on each gel, based on a protocol designed by the Wolfe lab [11,75,110]. Gels were assayed and analyzed in triplicate and a representative of each different age group was present on each gel. Protein transfer to PVDF membranes was performed. Membranes were blocked with Odyssey blocking buffer (LiCor, Lincoln, NE), diluted 1:1 (v/v) in Tris-buffered saline (TBS); incubated at 4°C in primary antibodies overnight with agitation, rinsed 3 times with TBS-Tween, and incubated in infra-red labeled secondary antibody. Bands were scanned with a LI-COR Odyssey Imager (Li-Cor Biosciences, Lincoln, NE). See Table 1 for antibody dilutions and sources.

### 2.5. Data analysis

**Fig. 2.**
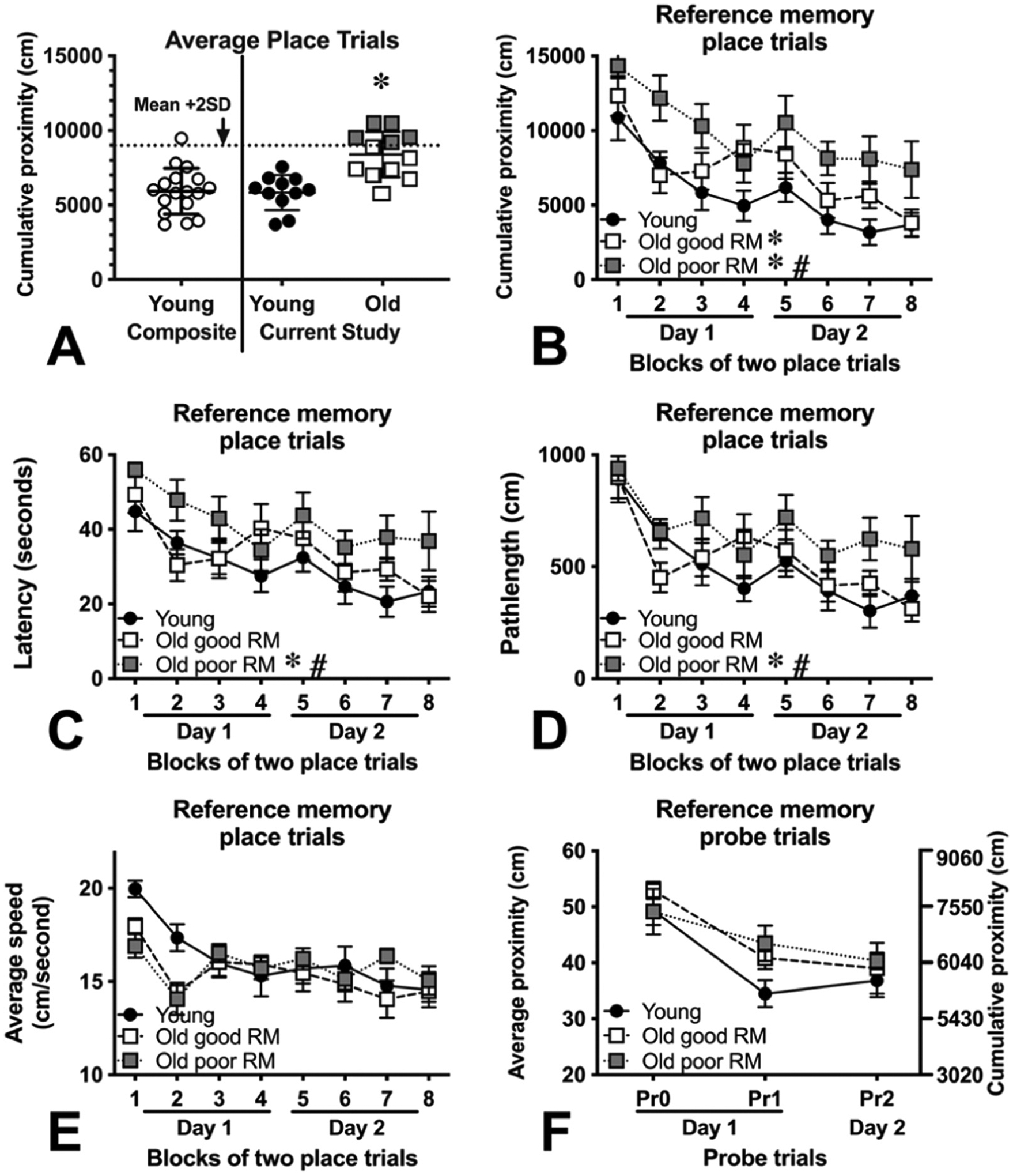
Behavioral testing graphs comparing cognitive functions in older male mice designated as having good or poor reference memory (RM) and young mice in reference memory tasks in the Morris water maze. A) Average performance across reference memory place trials for a composite of young mice from 2 different studies and the individual young and old male mice used in the current study. Older mice designated as Old poor RM (grey boxes) or Old good RM (white boxes) had averages greater or lesser, respectively, than composite young mean + 2 SD (dotted line). B-E) Cumulative proximity (B), latency (C), pathlength (D), and average speed (E) measures for Blocks of 2 place trials for reference memory. F) Average (left Y-axis) and cumulative proximity (right Y-axis) to the platform location in the reference memory probe trials. ANOVA and Fisher’s LSD post-hoc analysis. * indicates p < .05 for difference from young mice. # indicates p < .05 for difference from Old good RM mice. N = 11 young; 7 Old good RM, 5 Old poor RM. Pr = Probe. Data presented as mean ± SD (A) or SEM (B-F).

Behavioral testing data were analyzed as described previously [18]. Place, reversal and cued trials were assessed with cumulative proximity measures, which reflect search distances from the platform and were corrected for start position [32]. Average proximity measures were obtained for probe trials by dividing the cumulative proximity by the number of samples, which is always the same in probe trials (151 samples in 30 s) [32]. Because cumulative proximity is partially influenced by time to locate the platform and hidden platform trials can be up to 60 s, cumulative proximity in probe trials is not completely comparable to hidden platform trials in the place, reversal and cued trials. However, both the average and cumulative proximity scales are presented for probe trials. In line with the previous publication [121], the same mice were divided into two categories for good reference memory (good RM) and poor reference memory (poor RM) performers, based on acquisition performance in the place trials. This practice was in line with others [53,99,117], with some modification. The mice from the current study were combined with mice from a second study, which were tested similarly, in order to increase the N and thus the confidence in the divisions. The mean ± 1 standard deviation (SD) of cumulative proximity across all place trials for reference memory testing for young mice from the combined studies was 5923 ± 1530 cm (Fig. 2A, N = 17). The division between good and poor performers was based on 2 SDs above the mean of the young mice (5923 + (2 ×1530) = 8983 cm) and resulted in assigning 7 as old good RM mice and 5 as old poor RM mice (Fig. 2A). There was one young mouse with a score of 9469 cm. It was eliminated from the study due to a low N for young poor RM performers. Thus the young remaining under consideration were all categorized as good RM performers (within 2 SD of composite young mean) and will be referred to simply as “young”. For statistical comparisons, age/performance group effects will be used to refer to ANOVA results comparing young, old good RM and old poor RM mice. Because not all groups obtained the same level of performance at the end of the memory place trials, reversal place trial data are presented as both cumulative proximity to the new platform location and reversal trial data normalized to the last place trial blocks (the cumulative proximity to the new target within each reversal trial block minus the cumulative proximity to the target in the last place block of two trials). Positive scores indicate search farther from the target than in the last place blocks and negative values indicate a closer search in the reversal trials. Protein blots were analyzed using Li-Cor Odyssey software. For Western blotting, integrated intensity measures were obtained using median top/bottom background subtraction method with the use of Li-Cor Odyssey imaging software. Integrated intensity values for the known loads of caudal cortex were used to establish a linear fit standard curve with the use of Excel (Microsoft) and sample values were interpolated from the standard curve as caudal cortex equivalents. The standards were designed to reduce variability between blots and to interpolate from the linear portion of the standard curve. Both the splice cassette proteins and actin were interpolated from a standard curve for their respective proteins. Then the caudal cortex equivalent of the protein of interest was divided by the caudal cortex equivalent of actin in the same blot lane in order to control for loading. There were three technical replicates for each subject, brain region, and cell subfraction. The bands for each subject were quantified using the same sized selection area and outliers were discarded. Among all potential protein X brain region X subfraction samples (414 total), data from 46 frontal cortical and 11 hippocampal samples were unavailable due to insufficient sample, with the majority of these being from the S2 fractions. The material from this study originated from another study [121], which exhausted some of the samples. Data from an additional 9 frontal cortical and 11 hippocampal protein/subfraction samples were excluded due to blot analysis issues. Remaining Ns are indicated on the bar graphs. Statistical analyses were performed with repeated measures analysis of variance (RANOVA) for behavioral testing or one-way ANOVA for protein expression for separate proteins within each fraction, followed by Fisher’s least significant difference (LSD) using Prism software (Graphpad Software). Pearson correlation coefficients were generated for comparisons between protein expression and behavioral outcomes using Prism software.

## 3. Results

### 3.1. Behavioral testing

#### 3.1.1. Reference memory

**Fig. 3.**
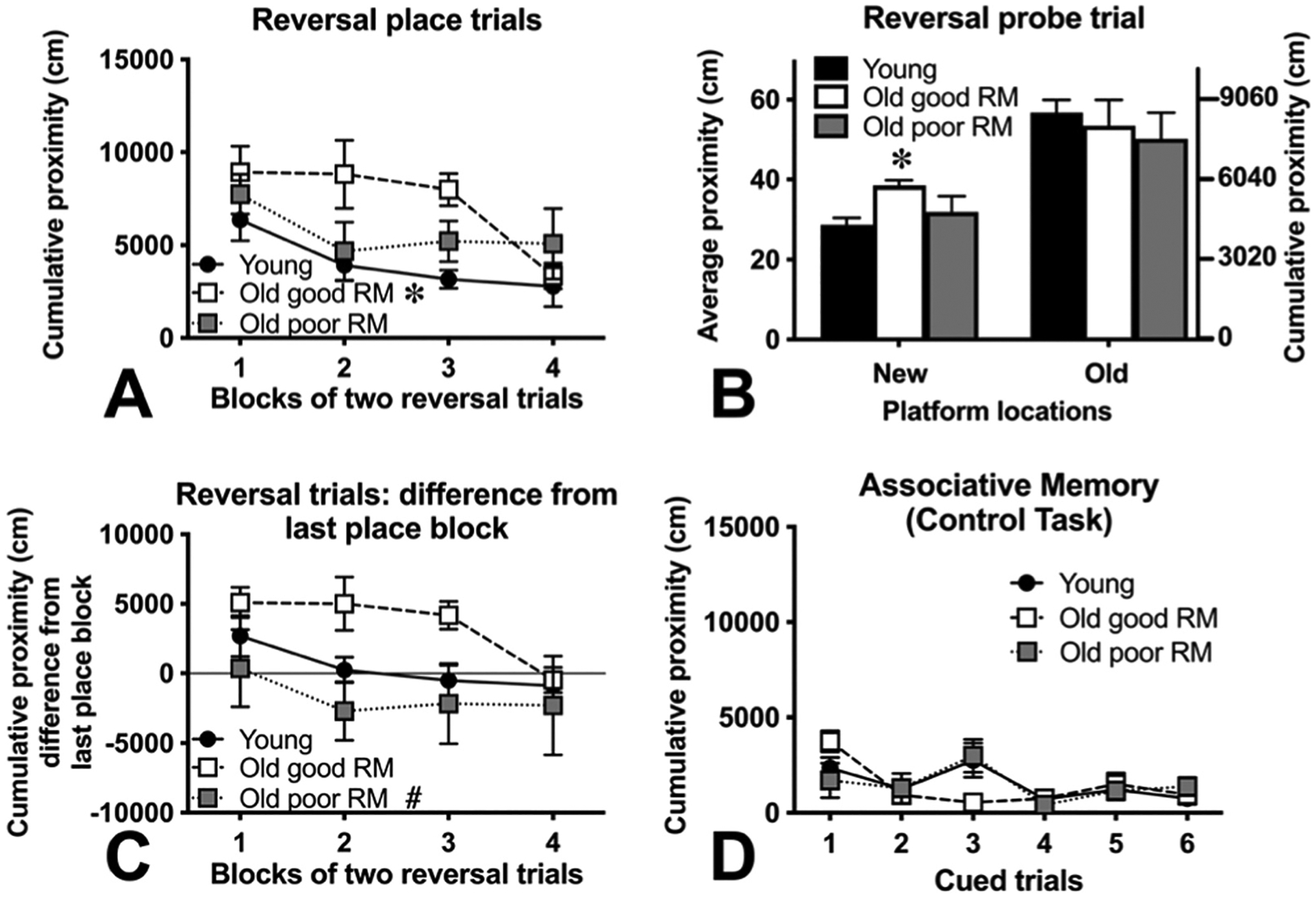
Behavioral testing graphs comparing cognitive functions in older male mice designated as having good or poor reference memory (RM) and young mice in reversal and cued tasks in the Morris water maze. Cumulative proximity (A) and difference in cumulative proximity from the average of the last two place block trials (C) in Blocks of 2 reversal trials. B) Average (left Y-axis) and cumulative proximity (right Y-axis) to the new and old platform locations in the reversal probe trials. D) Cumulative proximity to platform in the cued (control) trials. ANOVA and Fisher’s LSD post-hoc analysis. * indicates p < .05 for difference from young mice. # indicates p < .05 for difference from Old good RM mice. N = 11 young; 7 Old good RM, 5 Old poor RM. Pr = Probe. Data presented as mean ± SEM.

There was a significant main effect of age on cumulative proximity in the place trials for reference memory (F(1,21) = 20.2, p = .0002), with old mice having higher cumulative proximity than young (Fig. 2A). Individual performances for young (black circle), old good RM (open squares) and old poor RM (gray squares) are also indicated in Fig. 2A. After dividing the old mice into good and poor reference memory performers, there was a significant main effect of age/performance group on cumulative proximity (F(2,20)= 26.9; p < .0001; Fig. 2B), latency (F (2,20) = 11.7; p = .0004; Fig. 2C) and pathlength (F(2,20) = 10.3; p = .0008; Fig. 2C) in the place trials for reference memory. There was no main effect of age/performance on average speed (F(2,20) = .66; p = .53), but there was a near significant age/performance X trial block interaction (F(14,140) = 1.7; p = .055; Fig. 2E). Old poor RM had significantly higher cumulative proximities (p < .0001; Fig. 2B), latency to the platform (p < .0001; Fig. 2C), and pathlength (p = .0002; Fig. 2D) than young. Old poor RM also had significantly higher cumulative proximities (p = .0004; Fig. 2B), latency to the platform (p = .005; Fig. 2C), and pathlength (p = .003; Fig. 2D) than old good RM. Old good RM also had significantly higher cumulative proximities than young (p = .006; Fig. 2B), but did not differ from young in latency (p = .12; Fig. 2C) or pathlength (p = .42; Fig. 2D). There was also a significant main effect of trial block on cumulative proximity (F (7, 140) = 10.4; p < .0001; Fig. 2B), latency (F (7, 140) = 5.7; p < .0001; Fig. 2C), pathlength (F (7, 140) = 8.4; p < .0001; Fig. 2D), and average speed (F (7, 140) = 6.4; p < .0001; Fig. 2E) in the place trials for reference memory. The last block showed significantly lower values of these measures than the first block of two trials with data collapsed across age/performance groups (p < .0001; Fig. 2B–E). Due to the control trials occurring at the end of testing, the first block of reference memory place trials was also analyzed. There was no significant effect of age/performance group on cumulative proximity (p = .30; Fig. 2B), latency (p = .37; Fig. 3C) or pathlength (p = .95; Fig. 3D) on the first block of place trials.

There was no significant effect of age/performance group on average proximity in probe trials for reference memory (F(2,20) = 2.4; p = .12; Fig. 2C). There was a significant main effect of trial on average proximity in probe trials for reference memory (F (2, 40) = 12.7; p < .0001; Fig. 2C). Both the first (Pr1) and second (Pr2) test probe trials showed significantly lower average proximities than the naïve probe trial (Pr0), with data collapsed across age/performance groups (p < .0001; Fig. 2C). The equivalent cumulative proximity is shown on the right Y-axis (Fig. 2F), but is not entirely comparable to the place trials, due to a shorter maximum time in the probe trials.

#### 3.1.2. Reversal trials

There was a significant main effect of age/performance group (F (2,20) = 5.4; p = .014) and trial block (F (3,60) = 6.7; p = .0006) on cumulative proximity in reversal place trials (Fig. 3A). The old good RM mice had significantly higher cumulative proximity in reversal place trials than the young (p = .004), but old poor RM mice did not differ significantly from young (p = .16) or old good RM mice (p = .20; Fig. 3A). Cumulative proximity in trial block 4 was significantly lower than trial block 1 (p = .003; Fig. 3A). Due to the groups showing different performances at the completion of the reference memory place trials, the reversal trial scores were also normalized by subtracting individual cumulative proximity scores to the target in the last place trial block from the cumulative proximity to the new target in each reversal trial block (Fig. 3C). There was no significant main effect of age/performance groups on difference from last place block (F(2,20) = 3.2; p = .06), but the old good RM mice had higher normalized cumulative proximity than old poor RM mice (p = .023; Fig. 3C). There was a significant main effect of age/performance group on average proximity to the new platform position (F(2,20) = 5.8; p = .01), but no significant main effect of age/performance group on average proximity to the old position (F(2,21) = .37; p = .70) in the reversal probe trial (Fig. 3B). The old good RM mice had higher average proximities than the young in average proximity to the new position in the reversal probe trial (Fig. 3B).

#### 3.1.3. Cued Control Trials

There was no significant main effect of age/performance groups (F (2,20) = .05; p = .95), but there was a significant main effect of trial (F (5,100) = 5.1; p = .0003) on cumulative proximity in the cued trials (Fig. 3D). The last cued trial had a significantly lower cumulative proximity than the first trial (p = .0003; Fig. 3D).

### 3.2. GluN1 splice cassette expression

#### 3.2.1. Hippocampus

**Fig. 4.**
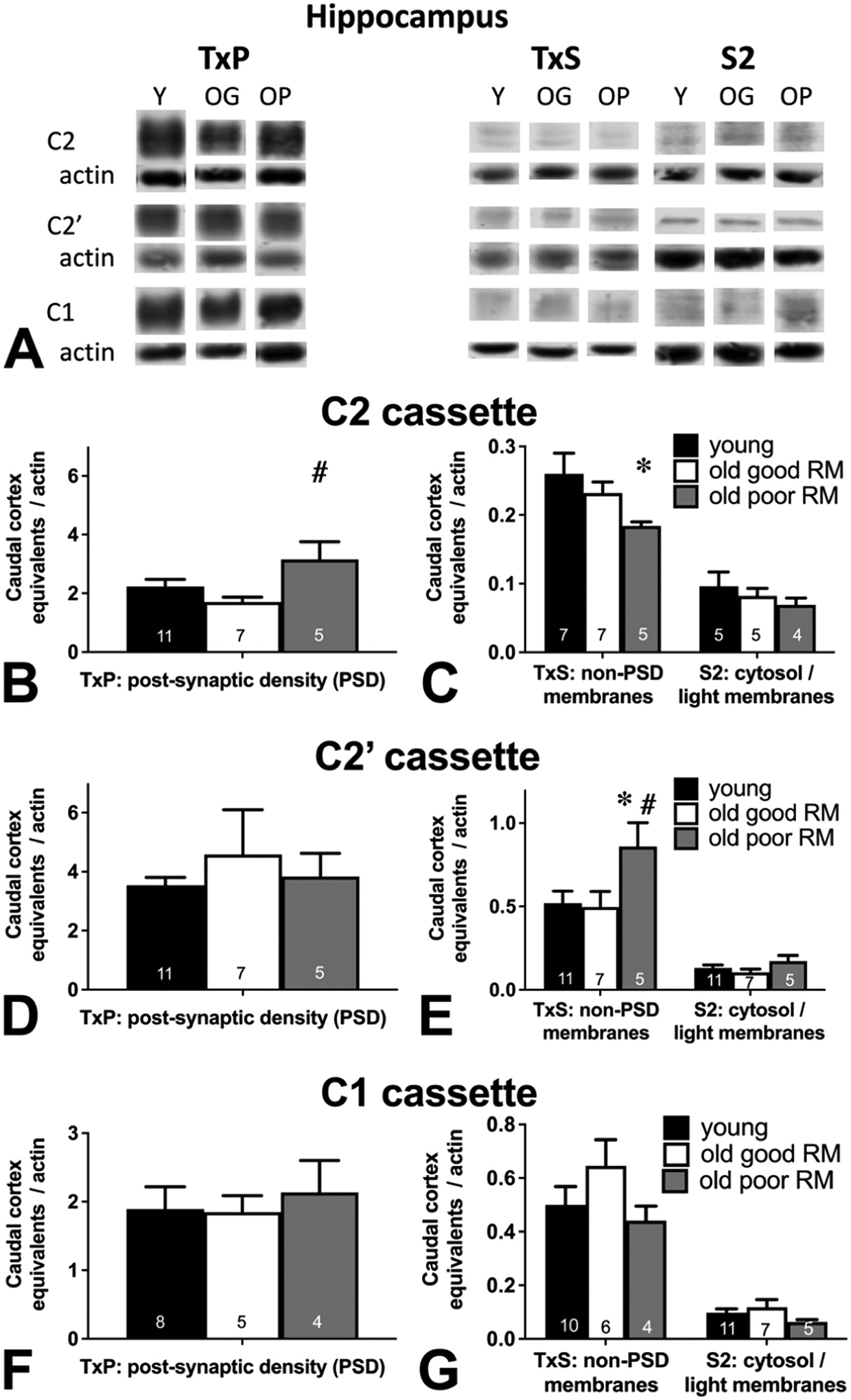
Western blot representative images (A) and graphs (B-G) for C-terminal splice cassettes (C2: B,C; C2’: D,E; and C1: F,G) in fractions (TxP: post-synaptic density, B,D,F; and TxS: non-PSD membranes and S2: cytosol/light membranes, C,E,G) from the hippocampus. ANOVA and Fisher’s LSD post-hoc analysis. * indicates p < .05 for difference from young mice. # indicates p < .05 for difference from Old good RM mice. Ns indicated in each bar. Y, young; OG, old good RM; OP, old poor RM.

There was a significant main effect of age/performance group on protein expression of the C2 cassette of the GluN1 subunit in the post-synaptic density (PSD; TxP fraction) of the hippocampus (F(2,20) = 4.0; p = .03; Fig. 4A,B). The old poor RM had significantly higher caudal cortex equivalents of C2 protein normalized to actin than old good RM (p = .01) and near significantly higher than young (p = .06; Fig. 4B). There were no significant main effects of age/performance group on protein expression of the C2 cassette in the non-PSD membranes (TxS fraction; F(2,16) = 2.7; p = .09) or cytosol/light membranes (S2 fraction; F(2,11) = .72; p = .51) in the hippocampus (Fig. 4A, C). However, the old poor RM group had significantly lower C2 cassette protein expression in the non-PSD membranes than young (p = .03; Fig. 4C). There was a significant main effect of age/performance group on protein expression of the C2’ cassette in the non-PSD membranes of the hippocampus (F(2,20) = 3.5; p = .05; Fig. 4A,E). The old poor RM mice had significantly higher C2’ cassette expression than both young (p = .03) and old good RM (p = .03) mice (Fig. 4E). There were no significant main effects of age/performance group on C2’ cassette protein expression in the PSD (F(2,20) = .40; p = .68; Fig. 4A,D) or cytosol/light membranes (F(2,20) = 1.8; p = .18; Fig. 4A,E). There were no significant effects of age/performance group on protein expression of C1 cassette of the GluN1 subunit in the PSD (F(2,14) = .15; p = .86; Fig. 4A, F), non-PSD membranes (F(2,17)= 1.4; p = .27; Fig. 4A,G), or cytosol/light membranes (F(2,20) = 1.55; p = .24; Fig. 4A,G).

#### 3.2.2. Frontal Cortex

**Fig. 5.**
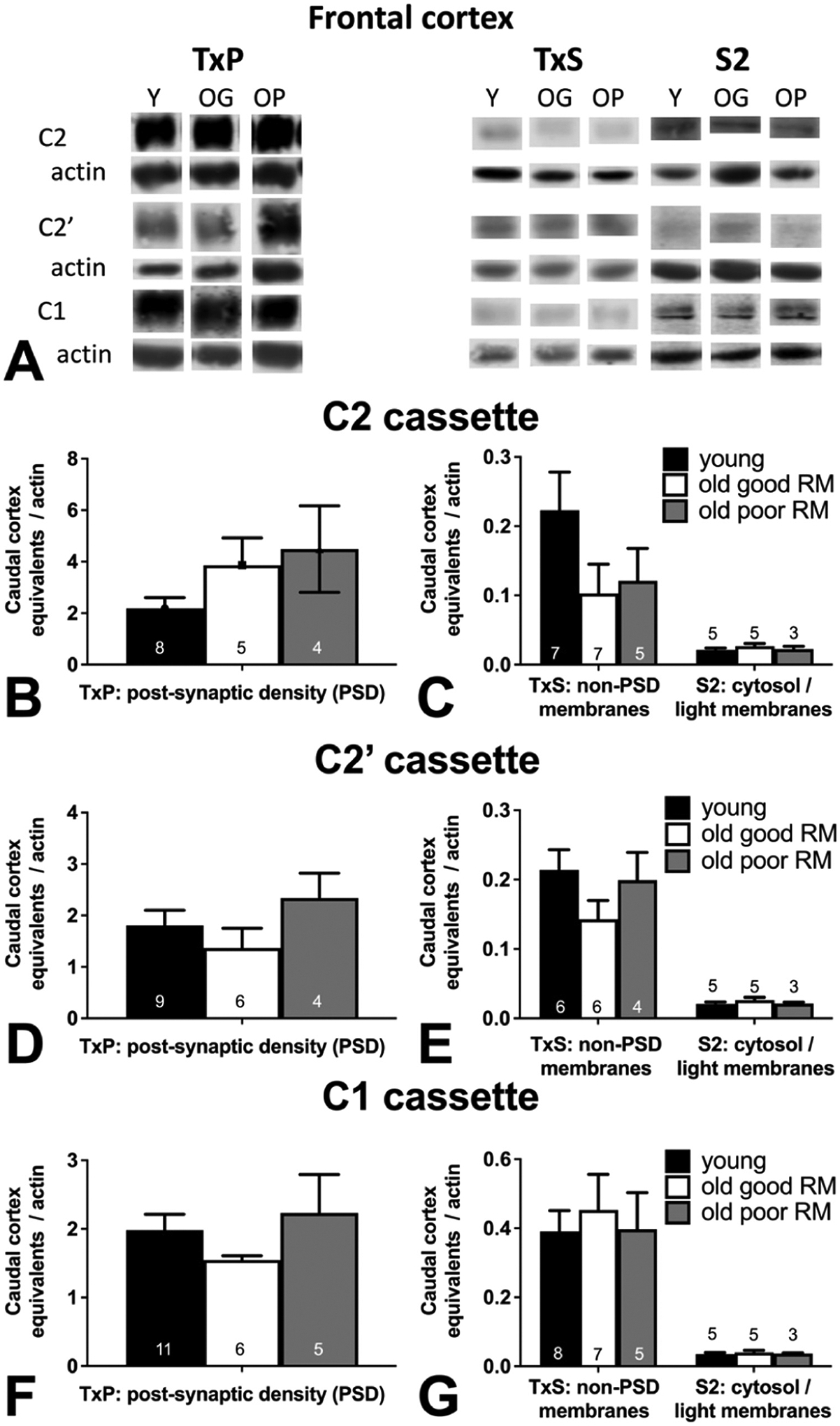
Western blot representative images (A) and graphs (B-G) for C-terminal splice cassettes (C2: B,C; C2’: D,E; and C1: F,G) in fractions (TxP: post-synaptic density, B,D,F; and TxS: non-PSD membranes and S2: cytosol/light membranes, C,E,G) from the frontal cortex. ANOVA and Fisher’s LSD post-hoc analysis. Ns indicated in each bar. Y, young; OG, old good RM; OP, old poor RM.

There were no significant effects of age/performance group on C2 cassette expression in the PSD (F(2,14) = 1.8; p = .20; Fig. 5A,B), non-PSD (F(2,16) = 1.9; p = .19; Fig. 5A,C), or cytosol/light membranes (F (2,10) = .69; p = .52; Fig. 5A,C) from the frontal cortex. There were no significant effects of age/performance group on C2’ cassette expression in the frontal cortex PSD (F(2,16) = 1.4; p = .29; Fig. 5A,D), non-PSD (F (2,13) = 1.6; p = .24; Fig. 5A,E), or cytosol/light membranes (F(2,10) = .93; p = .42; Fig. 5A,E). There were no significant effects of age/performance group on C1 cassette expression in the PSD (F(2,19) = 1.1; p = .36; Fig. 5A,F), non-PSD (F(2,17) = .16; p = .85; Fig. 5A,G), or cytosol/light membranes (F(2,10) = .24; p = .79; Fig. 5A,G) from the frontal cortex.

### 3.3. Correlational analysis

**Fig. 6.**
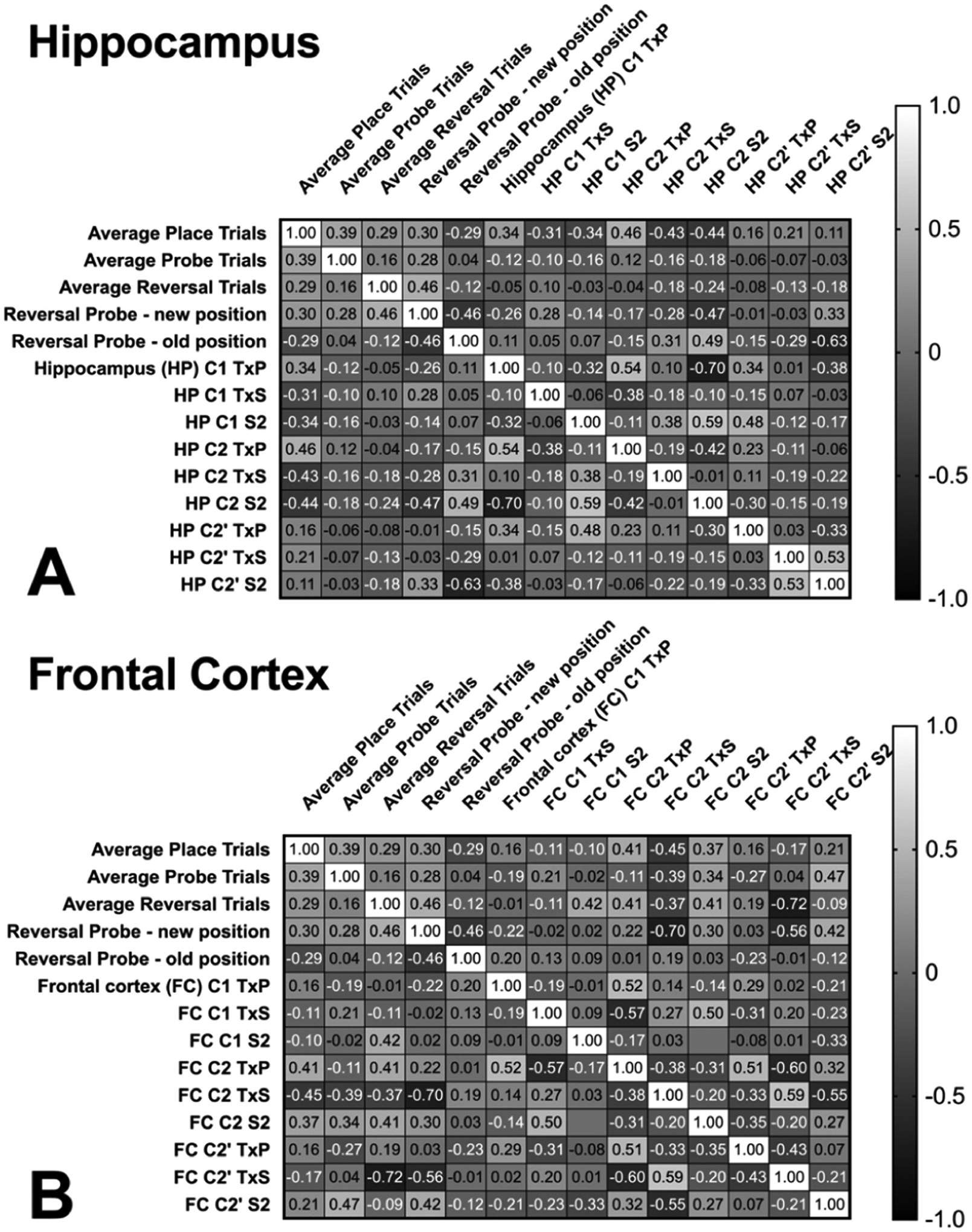
Correlation matrices of Pearson r values comparing behavioral outcomes and protein expression in different fractions from the hippocampus (A) and frontal cortex (B).

Correlational analysis was performed to explore the relationships between C-terminal cassettes of the GluN1 subunit and between cassettes and behavioral performances across all ages (Fig. 6). Due to multiple comparisons, a significance level of p ≤ .01 was applied.

#### 3.3.1. Relationships between cassettes

**Fig. 7.**
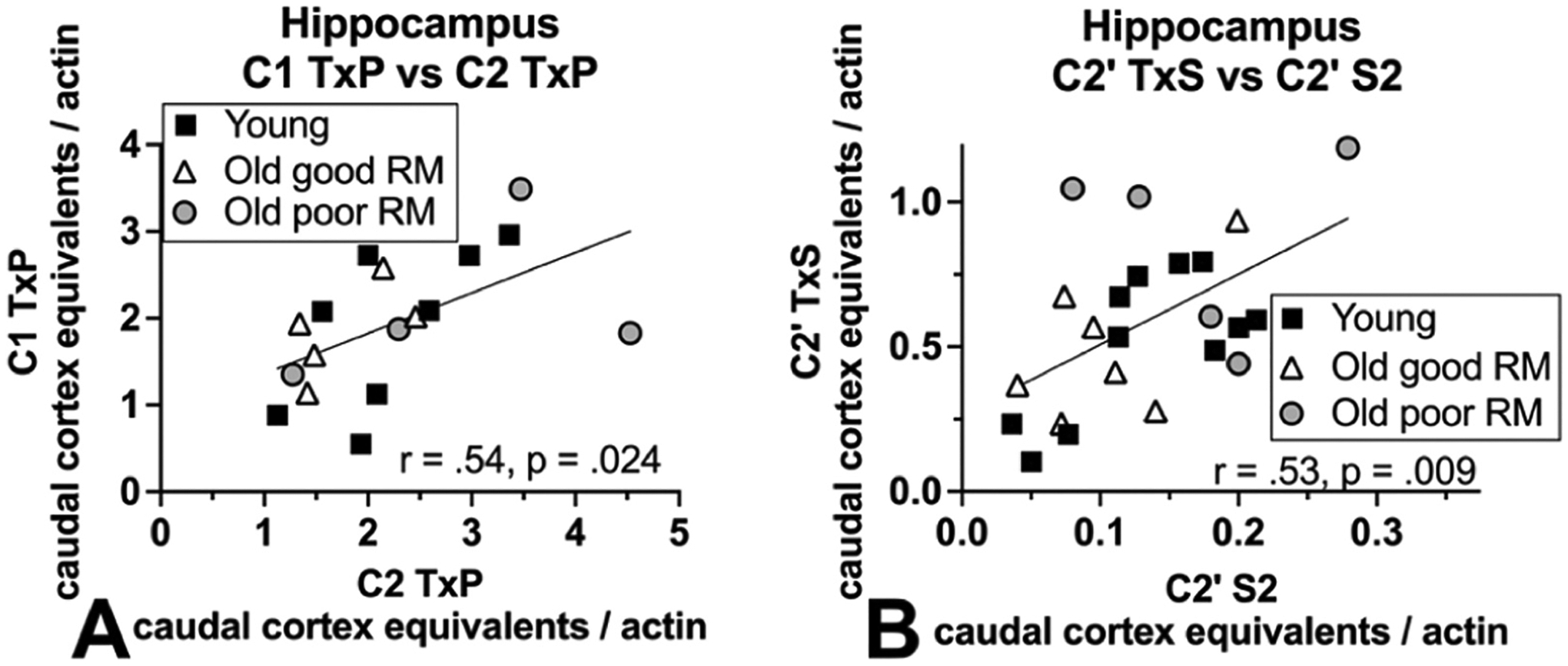
Correlation graphs for relationships between GluN1 cassettes in the hippocampus. A) There was a trend for a significant positive relationship between C1 and C2 cassettes in the TxP: synaptic membrane. B) There was a significant positive relationship between C2’ sequence in the TxS: non-PSD membrane and S2: cytosol/light membrane. Each symbol represents an individual animal. r, Pearson correlation coefficient; RM, reference memory.

There was a significant positive association in the hippocampus between expression of C2’ cassettes in the non-PSD membranes and cytosol/light membranes (r = .53, p = .009; Figs. 6A,7B). There was a trend for C1 cassette expression to show a positive relationship to C2 in the PSD in both the hippocampus (r = .54, p = .024; Figs. 6A,7A) and frontal cortex (r = .52,p = .03, Fig. 6B).

#### 3.3.2. Relationships between cassettes and behavior

**Fig. 8.**
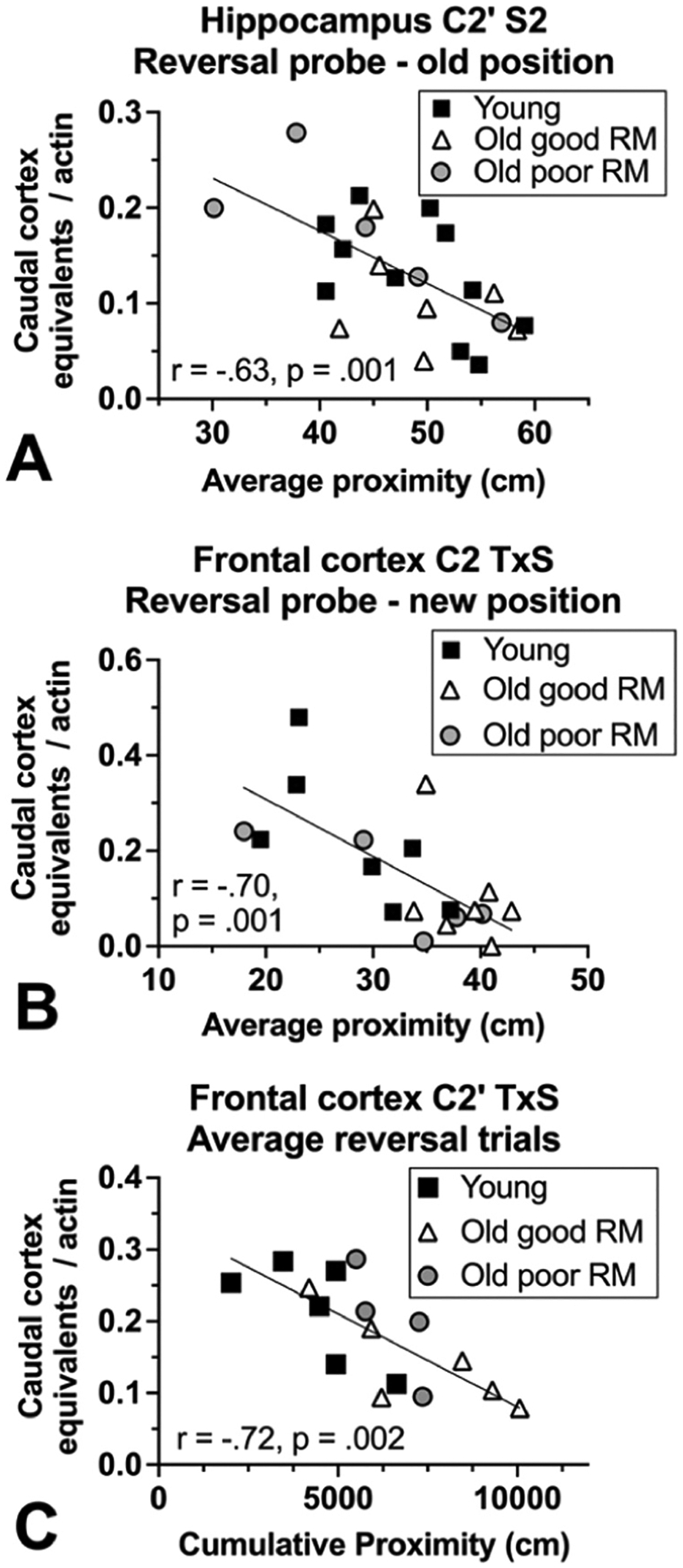
Correlation graphs for relationships between GluN1 cassettes in the hippocampus (A) or frontal cortex (B,C) and performance in reversal trials across all age/performance groups. A) Hippocampal C2’ sequence in the S2 fraction was negatively correlated with average proximity to the old platform location (lower average proximity suggests perseveration) B) Frontal cortex C2 cassette and C2’ sequence in the TxS showed a negative relationship to proximity to the new platform location in the reversal probe trial (B) and average place reversal trials (C) (lower average proximity indicates good performance). C) Symbols are individual animal results. r, Pearson correlation coefficient; RM, reference memory.

In the hippocampus there was a significant negative relationship between levels of C2’ cassette in the cytosol/light membranes and average proximity to the old platform location in the reversal probe trial (r = −.63, p = .001; Figs. 6A, 8A). This indicates that mice with higher levels of C2’ cassette showed closer proximity to the old platform location. In the frontal cortex, there was a significant negative relationship between levels of C2 cassettes in the non-PSD fraction and average proximity to the new platform location in the reversal probe trial (r = −.70, p = .001; Figs. 6B,8B). Also in the frontal cortex, the C2’ protein in the non-PSD fraction showed a negative relationship with cumulative proximity in the reversal platform trials (r = −.72, p = .002; Figs. 6B,8C). In both cases, mice with higher levels of C2 or C2’ protein in the extrasynaptic membrane spent more of the search closer to the new platform location.

## 4. Discussion

The main findings from this study were that old mice with poor reference memory showed the most changes in GluN1 C-terminal cassettes in the post-synaptic density and extrasynaptic membrane from the hippocampus. The C2 cassettes were increased in the synapse and decreased in the extrasynaptic membrane compared to old mice with good reference memory or young, respectively. C2’ cassettes were increased in the extrasynaptic membrane in old mice with poor reference memory, as compared to both young and old good performers. Across age groups, the GluN1 cassettes showed significant relationships with cognitive flexibility trials.

As previously reported [121], these old mice with good reference memory performed worse than young mice overall in the acquisition phase, but the old mice with poor memory were significantly worse than both old good RM and young, with respect to cumulative proximity measures. Using a similar method in aged rats to identify impaired versus unimpaired [32] or a median split to designate aged humans as good or poor performers [98,124], results showed similar performances between the young and good performers. There were no significant differences in the probe trials in the current study, so the deficit in this study was primarily in the acquisition of memories (i.e., during learning). Part of the issue with no differences between groups in probe trials was the young regressing on the end of day 2 compared to the end of Day 1, but the old mice also didn’t show any group differences like they did in the learning trials. We used 2 days of massed trials (2-minute intertrial interval), with a 1-hour break between sets of 4 place trials and a 1-hour break between the last place trial and probe trial. Memory retention is reduced in massed trials, as compared to spaced trials [8]. There is also a reduction in selective search in probe trials as the delay to probe trial increases from 10 min to 4 h [8]. In our laboratory, 12 days of training is more likely to show significant age-related impairments in both place and probe trials [61,17] than 2 days [121,123], so it is possible that the training was too short for them to develop a preference for the location when the platform was missing. In support of this is that they would have only had 1 night of sleep for memory consolidation [97,67]. Mice show declining preference within the last 10 s of a 30 s probe trial [17]. It is possible that with only two days of training, this might occur earlier in the probe trial. There was some evidence of memory formation across all the groups, however, with improvement from the naïve trial in both post-learning probe trials 1 and 2.

The old mice with good reference memory performance were significantly impaired in both the place and probe trials in the reversal task, showing a deficit in cognitive flexibility [45,51]. They differed from young in absolute cumulative proximity and their performance was even more strikingly different from old poor RM mice when the data was normalized to the performance at the end of the memory place trials. Based on the probe trial proximity to the old platform site, they did not appear to be perseverating on the old location, so it may have been a loss of confidence in the permanence of a new position. This ability to acquire a task well, but perform worse in a reversal task has been seen in female rats exposed to chronic stress neonatally [41]. The old poor learners performed similarly to young in the reversal trials. It is possible that the old poor RM mice didn’t learn the initial location well enough to interfere with acquiring a new location. However, they showed closer proximities to the new location than they had in the reference memory place trials, as shown by reversal trial performance normalized to the last two trials of the memory place trials, so it wasn’t that they had plateaued in the acquisition phase. There did not appear to be any visual, motor or motivational problems during testing, based on no significant group differences in cued trials or the first block of place trials. Cued trials were performed last due to previous experience that young mice do not show a learning curve in the place trials when cued trials are conducted first [64].

This study relied primarily on proximity measures to assess water maze performance. This was partially based on older C57BL/6 mice typically displaying decreased locomotor activity and slower swim speeds [95,115], which makes latency measures problematic to interpret. In this study, however, there were no significant differences in swim speed. Nonetheless, proximity measures have been identified as the most sensitive measures for the water maze [57]. In both the present study and a virtual Morris water maze study in humans [124], the proximity measures were able to identify more significant differences than either latency or pathlength. These latter measures did not show the significant differences between old good learner mice and young that were seen with cumulative proximity scoring in this study.

The validation of the subfractionation technique in this study has been demonstrated in a previous publication [121], which used the same animals and tissues that were used in the present study. Blots in that prior paper demonstrate enrichment of post-synaptic density protein 95 kDa (PSD-95), NMDAR subunits GluN2A and GluN2B, and Fyn in the TxP and higher protein expression for p97ATPase and striatal-enriched protein tyrosine phosphatase (STEP) in the TxS and S2 fractions. Similar results have been seen in other subfractionation studies [33,47]. STEP is associated with extrasynaptic NMDARs [113]. GAIP-interacting protein, C terminus (GIPC), which has been shown to be primarily excluded from the synaptic membrane and preferentially associated with extrasynaptic NMDARs [118], was enriched in TxS and S2, compared to TxP. This suggests that our subfractionation technique was effective.

The hippocampus is a very important brain region for spatial memory [83,48]. Lesions of the hippocampus cause problems with both working and reference memory [81,90]. There is evidence of declines in hippocampal functions, including spatial memory, in both primates and rodents during aging [123,32,6,31]. NMDARs are in high density in the hippocampus [62,60] and play important roles in synaptic transmission and spatial memory [2,12,37,76,80,82]. Long Term Potentiation (LTP) is one mechanism believed to subserve memory at the synaptic level [4, 21,80,82,14]. Long-Term Depression (LTD) is also a form of synaptic plasticity, but it inhibits synaptic transmission [68,85]. NMDARs initiate LTP and LTD in the hippocampus [79,55,80,14,7,36]. Given that the GluN1 subunits are necessary components of NMDARs, alterations in the splice cassettes in the hippocampus with increased age are likely to significantly impact synaptic plasticity and memory.

**Fig. 9.**
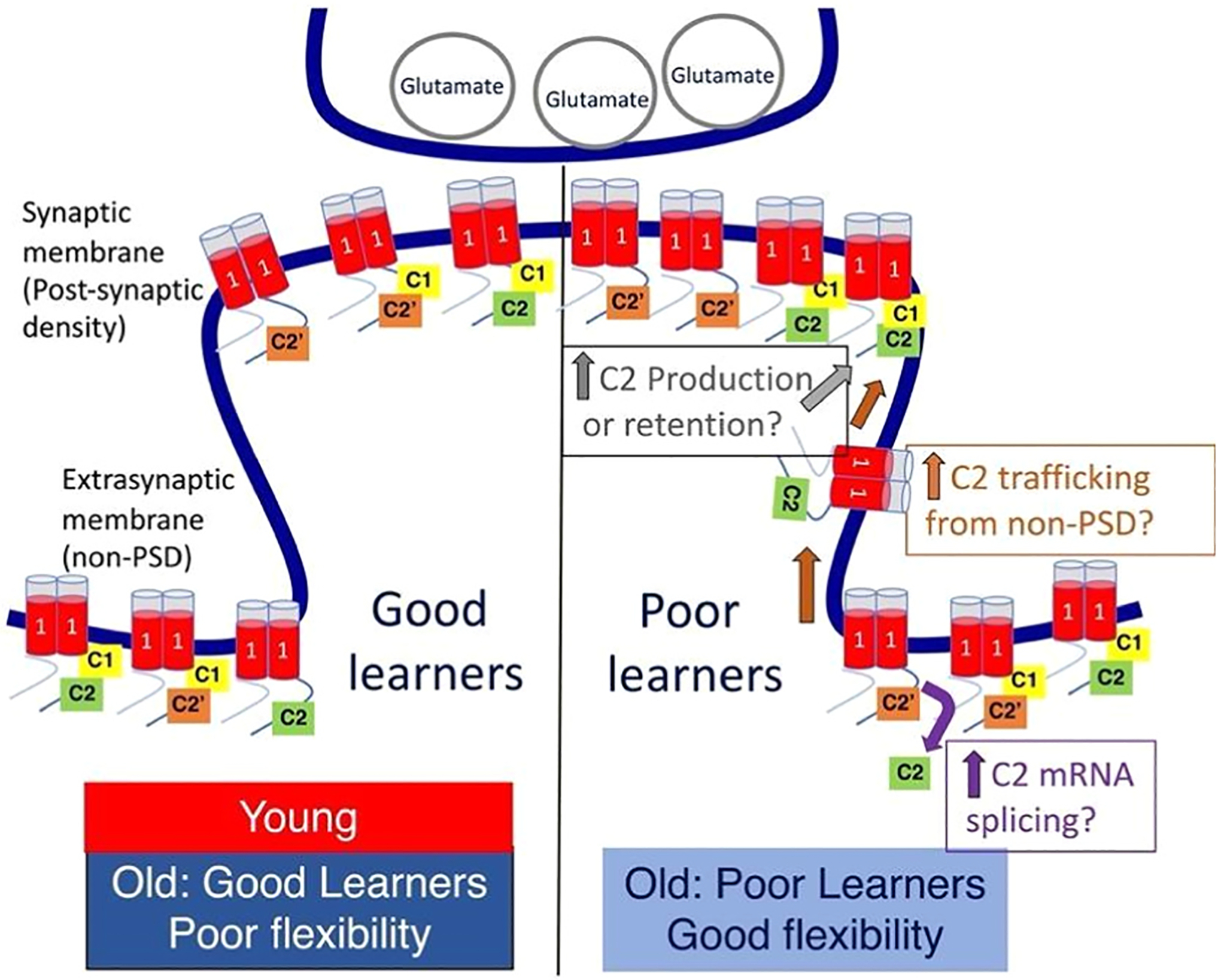
Older mice with good acquisition of reference memory, but poor cognitive flexibility, showed similar splice cassette levels to young mice in the hippocampus. Older poor learners with good flexibility showed increased expression of C2 cassettes in the synaptic membrane and decreased expression in the extrasynaptic membrane, which could be due to increased production, retention, or trafficking from the extrasynaptic membranes. C2’ cassettes were increased and C2 protein was decreased in the extrasynaptic membranes, which could be due to enhanced splicing of C2 cassettes. 1 = GluN1 subunit, The blue subunits behind the red subunits represent GluN2 or GluN3 subunits making up tetramer NMDARs.

The significant changes in C-terminal cassette expression were confined to the hippocampus of the old mice with poorer reference memory. C2 cassette protein levels were increased in the synaptic membrane compared to old mice with good learning ability and were decreased in the extrasynaptic membrane compared to young (Fig. 9). This could be due to increased trafficking of C2-containing NMDARs from extrasynaptic to synaptic membranes (Fig. 9). There is evidence that NMDARs exocytose into the membrane along the dendrite or on the side of spines [40,92,111] and laterally diffuse into the synaptic membrane [107,71]. Although caudal cortex homogenate was used as the standard for all subfractions, the total protein and actin levels used to normalize the proteins of interest may have differed. There was a larger increase from young in both amount and percent for the C2 cassette in the synaptic membrane (37.5 %) than the decrease from young in the extrasynaptic membrane (29 %). This suggests that the change is unlikely to be fully explained by trafficking. There could be increased production of C2-containing NMDARs destined for the synaptic membrane or increased retention on synaptic membranes (Fig. 9). Enhanced synaptic activity tends to increase expression of the C2 cassette [84]. The accumulation of more C2-containing NMDARs on old poor learner’s synaptic membrane compared to old good learners may reflect the culmination of more receptors reaching the plasma membrane due to higher levels of production. Across aging in this study, the levels of C2 showed a trend for association with the C1 cassette in the hippocampal synaptic membrane, suggesting that the GluN1–1 variant predominated. Increased synaptic activity enhances cleavage of the C1-containing tail of the GluN1–1 for translocation to the nucleus [125]. Conversely, reduced synaptic activity leads to increased localization of NMDARs on synaptic membranes [96]. So, the increase in C2 levels in the synaptic membrane of the old poor RM mice might reflect a decrease in synaptic activity, leading to both the retention of NMDARs on synaptic membranes and GluN1 subunit C1-C2 tails.

It is also possible that the decline in C2 protein in the extrasynaptic membrane of old poor learners was due to increased splicing of the C2 cassette leading to the increase in the expression of the C2’ sequence that was also seen in the extrasynaptic membranes of these mice (Fig. 9). Enhanced synaptic activity tends to increase expression of the C2 cassette, whereas decreased or blocked activity promotes splicing of C2 to the alternate sequence C2’ [84]. It’s not clear whether localized NMDAR activity at the synaptic or extrasynaptic membranes could produce these changes in splicing and then target them to the appropriate membrane or whether these changes can both be explained by decreased activity (see above about retaining C2). The presence of C2’ enhances surface expression of NMDARs, compared to C2 [39]. The significant positive correlation between extrasynaptic and cytosol/light membrane C2’-containing NMDARs suggests that mRNA splicing is enhanced and these receptors are trafficked and exocytosed to the extrasynaptic membrane, but stall out and build up there rather than diffusing to the synaptic membrane in the old poor learners.

In the frontal cortices of C57BL/6 mice across aging, increased association of post-synaptic density protein-95 (PSD-95; a scaffolding protein that holds NMDAR to the synaptic membrane [113]) with GluN2B-containing NMDARs was related to poorer performance in a reference memory task [120]. Nonsignificant trends for increases in GluN2A and p1472GluN2B in the synaptic membrane of the hippocampus were seen in the same old poor RM mice [121] that were used in this study, suggesting that an increase in NMDARs in the synaptic membrane may be detrimental to reference memory. This is in line with other evidence that the NMDAR in older mice and rats is not as beneficial for memory as in younger individuals [88,106,123,43,46,65,89]. C2-expressing NMDARs are necessary for stabilizing spine density in the hippocampus [3], so it is not clear why enhancement of these receptors was associated with poorer memory. The old good learners in this study appeared to maintain similar levels of C-terminal splice cassettes in the hippocampus to young mice.

There were no significant effects of age/performance groups on C-terminal splice cassettes in the frontal cortex in this study. Although in the same mice in a previous report, there were age-related declines in GluN2B in the synaptic membrane of old mice from both the good and poor memory groups and an increase in Fyn, a kinase that phosphorylates GluN2B subunits [103,108], in old poor RM mice in the synaptic membrane of the frontal cortex [122]. A prior report from our laboratory showed significant declines during aging in both C2 and C2’ protein in crude synaptosomes from frontal cortex [17]. The subfractions in the current study were produced from crude synaptosomes, but the processing (centrifuge speed and time, addition of Triton X-100) was different from [17]. The oldest mice were older (26 vs. 24 months) and behavioral testing was more extensive (25 vs 6 days) in the Das, et al. study [17], although there were no significant differences between naïve and behaviorally characterized mice in that study.

Across all ages, the significant relationships for C-terminal splice cassette proteins were with reversal probe or place trials. In the hippocampus, higher amounts of C2’ containing NMDARs in the cytosol/light membranes were associated with perseveration to the old platform location in the reversal probe trial. In the frontal cortex, higher C2 or C2’ protein levels in the extrasynaptic membrane were associated with better cognitive flexibility in the reversal probe or place trials, respectively. This is consistent with NMDARs playing a role in cognitive flexibility [10,15,66,23,38,104,20].

In conclusion, this study provides evidence that older C57BL/6 mice can be separated into good and poor reference memory groups based on the acquisition phase in the Morris water maze. The old poor learners showed increased levels of the C2 protein in the synaptic membrane and the C2’ protein in the extrasynaptic membranes in the hippocampus. The same proteins in the extrasynaptic membrane from the frontal cortex were associated with good cognitive flexibility. Thus, although alternative splice forms of the GluN1 subunit appear to contribute to cognitive declines during aging, the complexity of these changes and relationships suggest that interventions involving manipulating splicing of the C-terminal tail of the GluN1 subunit would likely exacerbate memory or cognitive flexibility problems or both in aged individuals.

## Supplementary Material

1

## Figures and Tables

**Table 1:** Antibody dilutions and sources for Western blots.

NMDA Receptor NR1 Subunit, Splice Variant C1 (Host: Rabbit)	1:500	Phosphosolutions	Cat. 1505-C1
NMDA Receptor NR1 Subunit, Splice Variant C2 (Host: Rabbit)	1:1000	Phosphosolutions	Cat. 1506-C2
NMDA Receptor NR1 Subunit, Splice Variant C2’ (Host: Rabbit)	1:1000	Phosphosolutions	Cat. 1507-C2’
Actin (Host: Mouse)	1:250	Santa Cruz	sc–47778
Secondary Antibody - Alexa Fluor 680 Goat-anti-Rabbit IgG	1:4000	Rockland	611–144–003
Secondary Antibody - IRDye^®^ 800CW Donkey anti-Mouse IgG	1:5000	Li-Cor Biosciences	

## Data Availability

Data will be made available on request.

## Appendix A. Supporting information

Supplementary data associated with this article can be found in the online version at doi:10.1016/j.brainresbull.2025.111502.
