# Supplementary material for: Higher levels of GluN1 splice cassettes, C2 and C2’, in hippocampus of aged mice were associated with poor spatial reference memory
Source: Brain Res Bull. Author manuscript; Available in PMC 2026 Jun 16. (PMC13271759; doi:10.1016/j.brainresbull.2025.111502)
Supplement: 1 [file NIHMS2180938-supplement-1.pdf]

Supplemental Figures. *Western blots that the band images in Figures 4 and 5 were obtained from. Please note that in some cases the gel was cut in order to stain for GluN1 splice cassettes and actin on separate blots. Fraction, brain region and GluN1 splice cassette is indicated on the top of each image.*

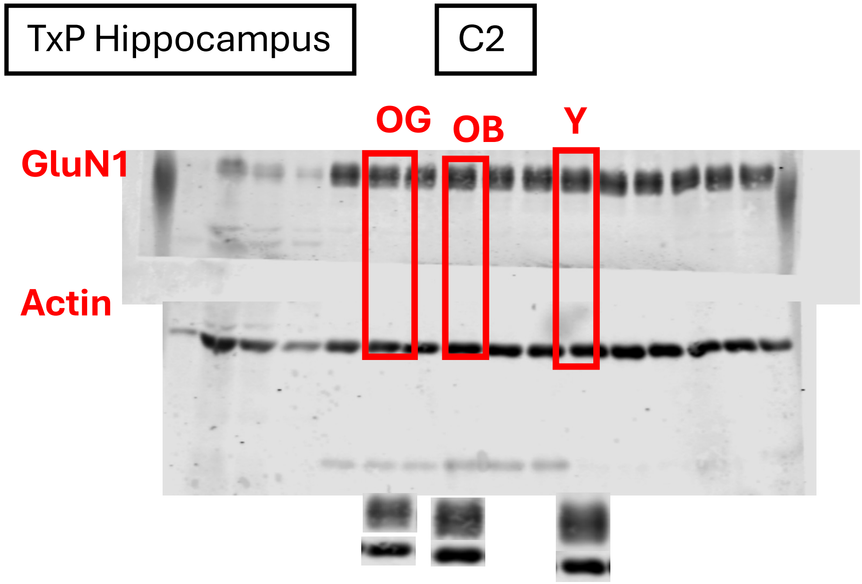

TxS
Hippocampus

C2

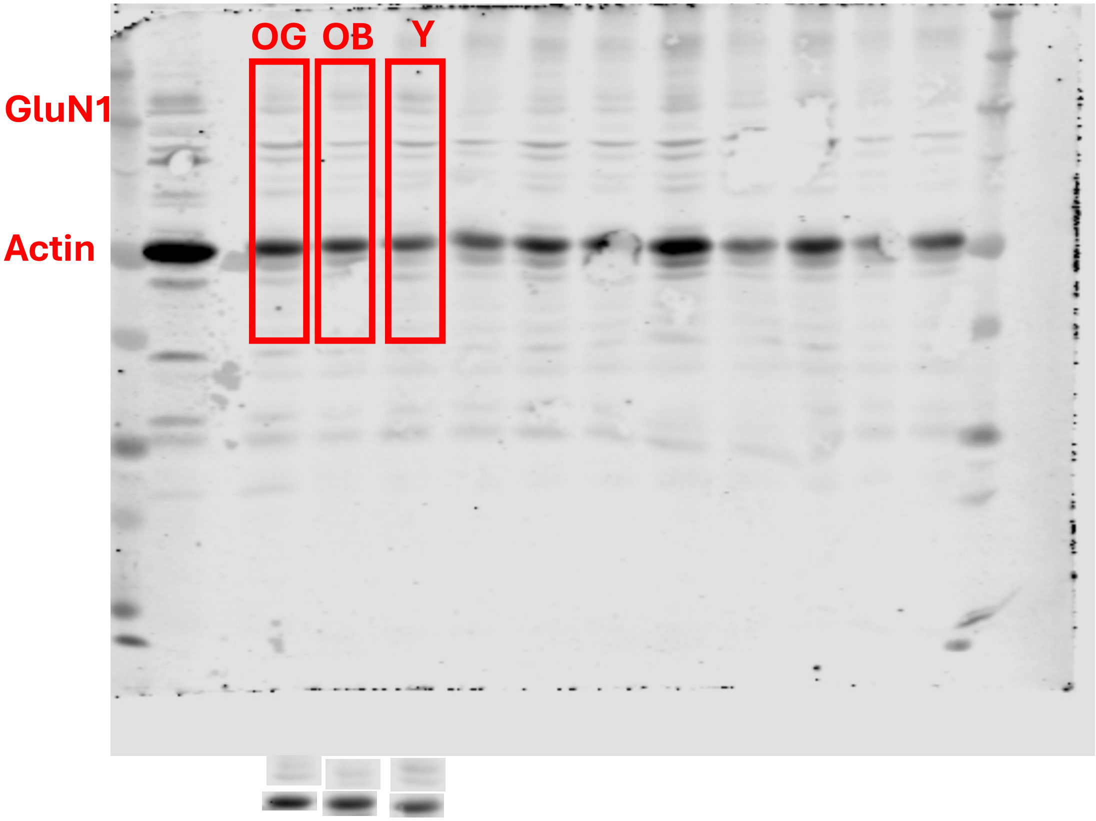

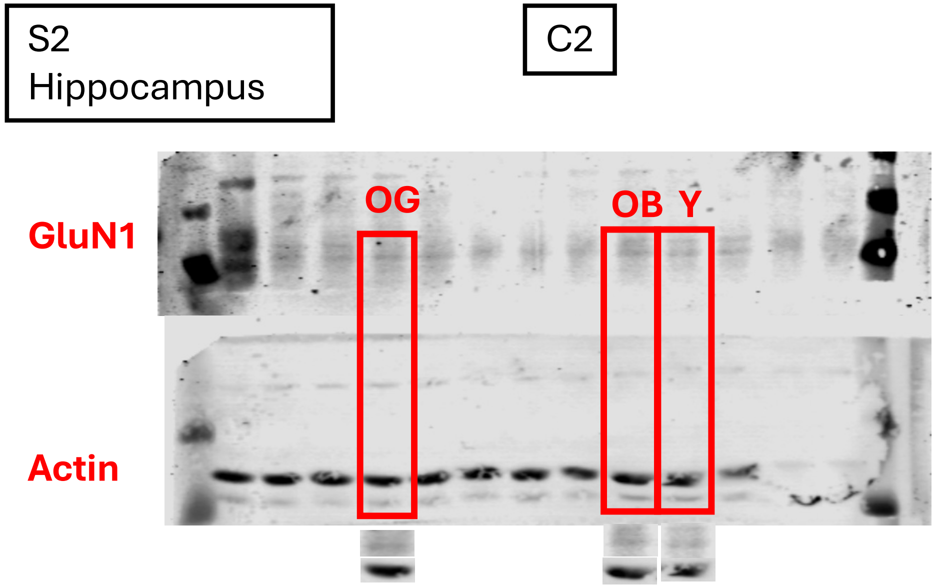
S2
Hippocampus
C2
GluN1
Actin
OG
OB
Y

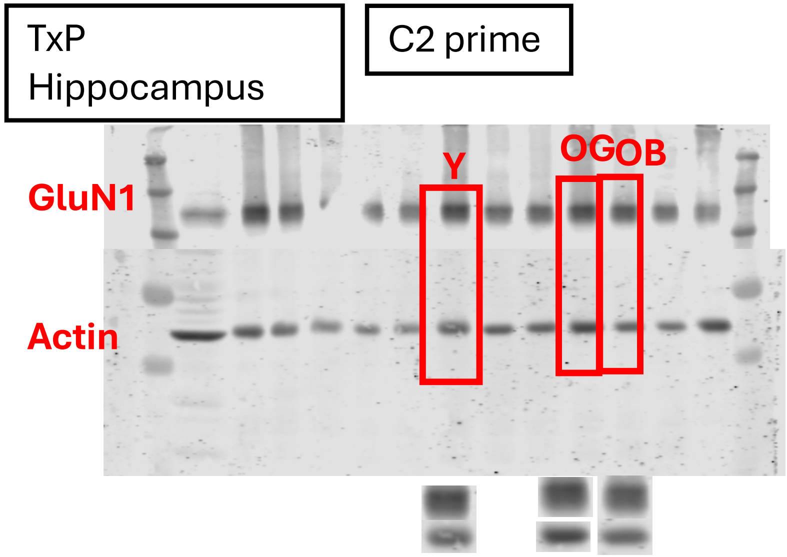
TxP
Hippocampus
C2 prime
GluN1
Actin
Y
OG
OB

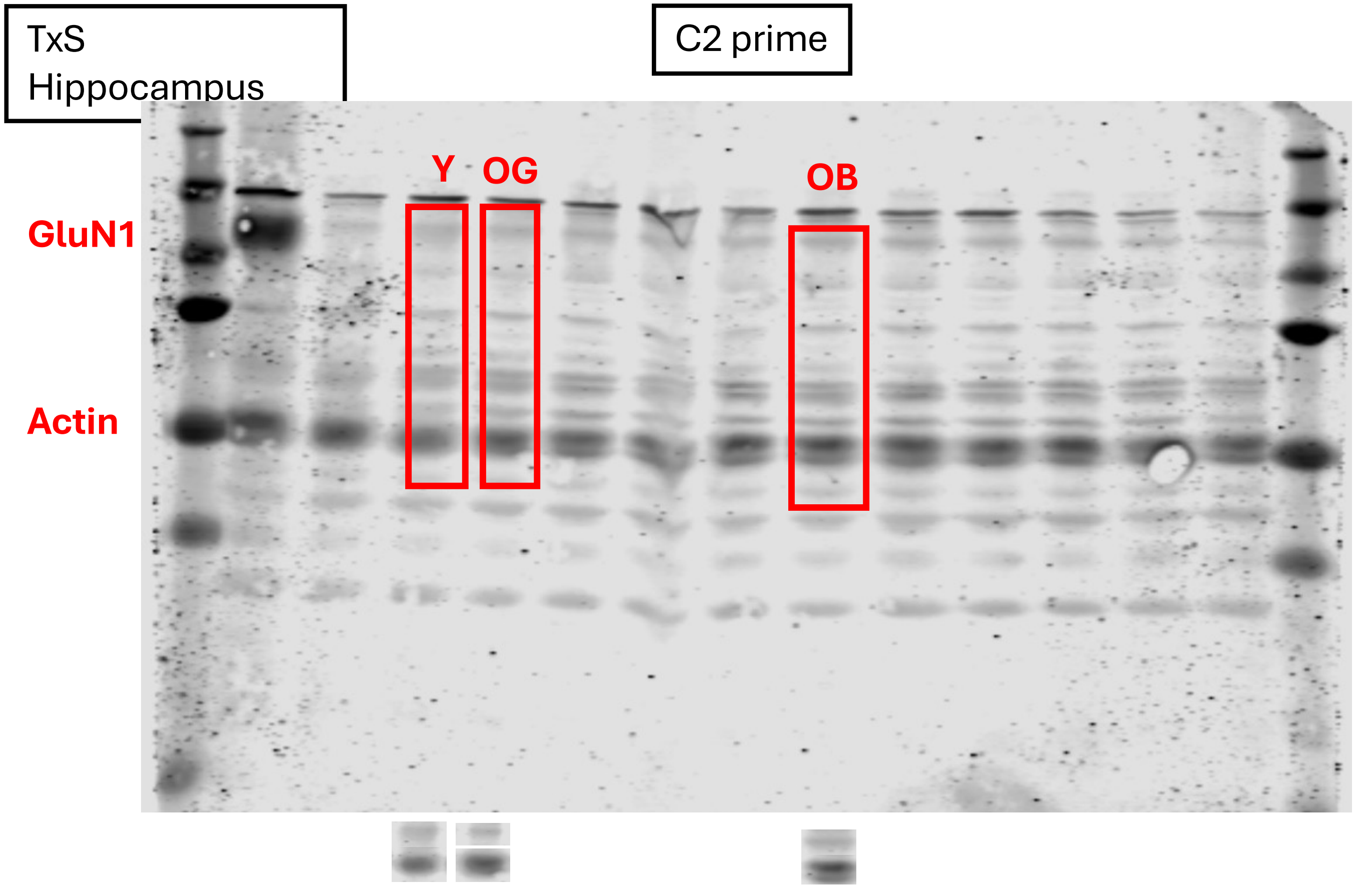

This is a demonstration blot in which several targets were probed, including GluN1-C2’ and Actin.

S2
Hippocampus

C2’

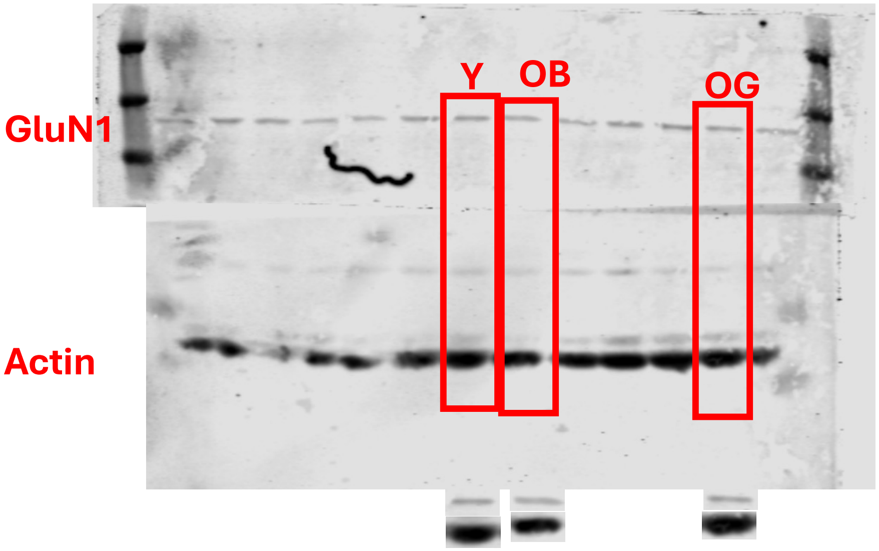

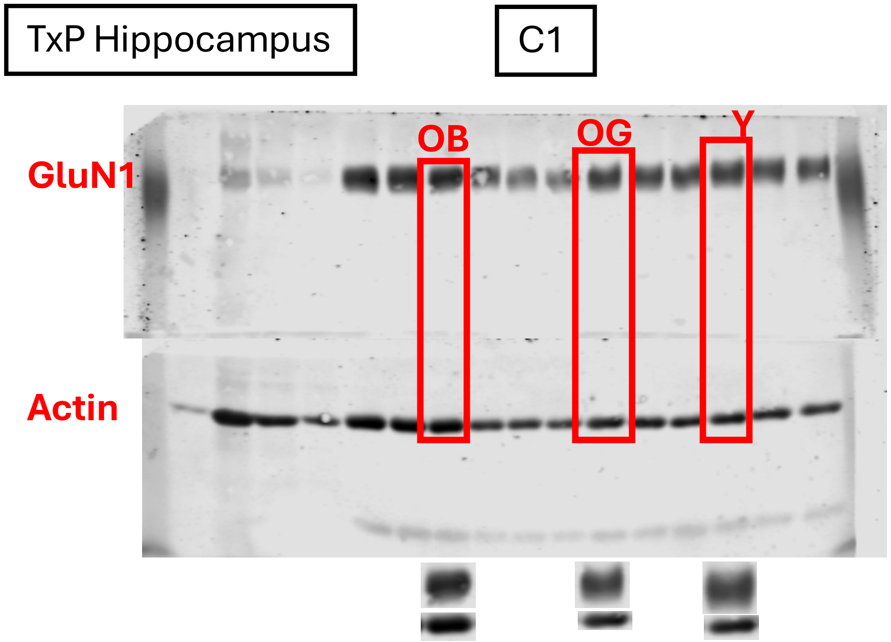
TxP Hippocampus
C1
GluN1
Actin
OB
OG
Y

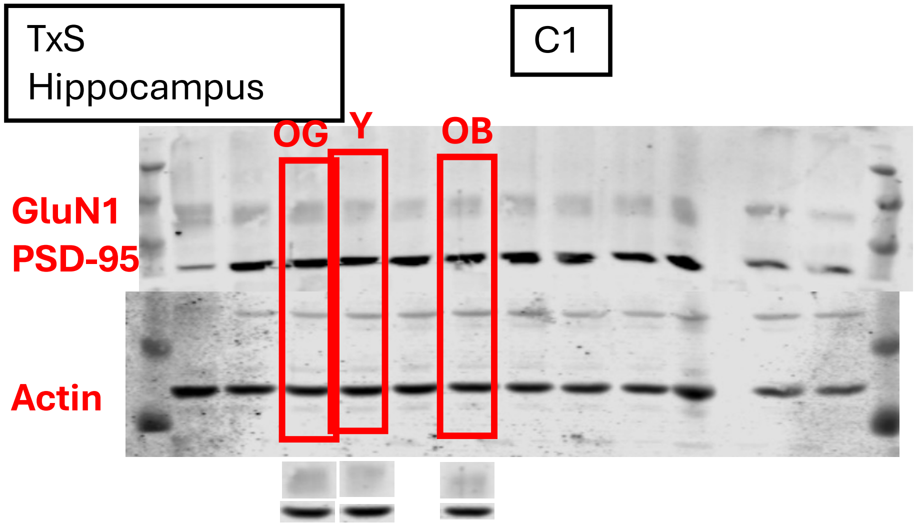
TxS
Hippocampus
C1
OG
Y
OB
GluN1
PSD-95
Actin

S2
Hippocampus

C1

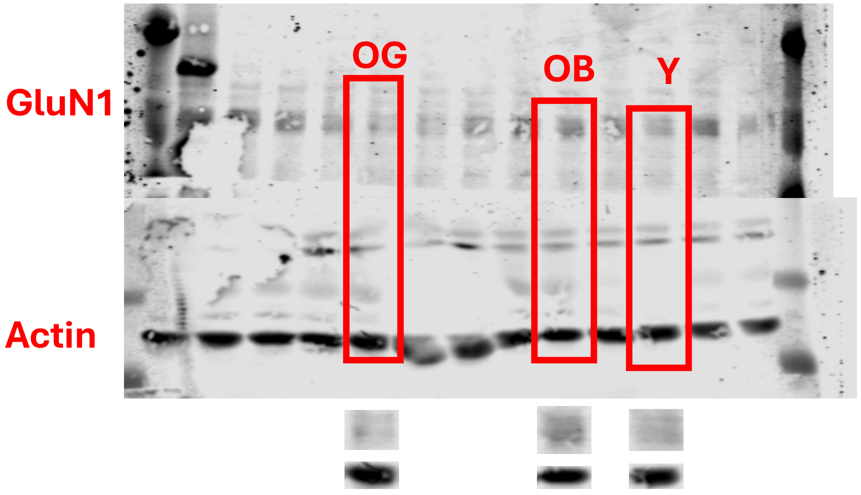

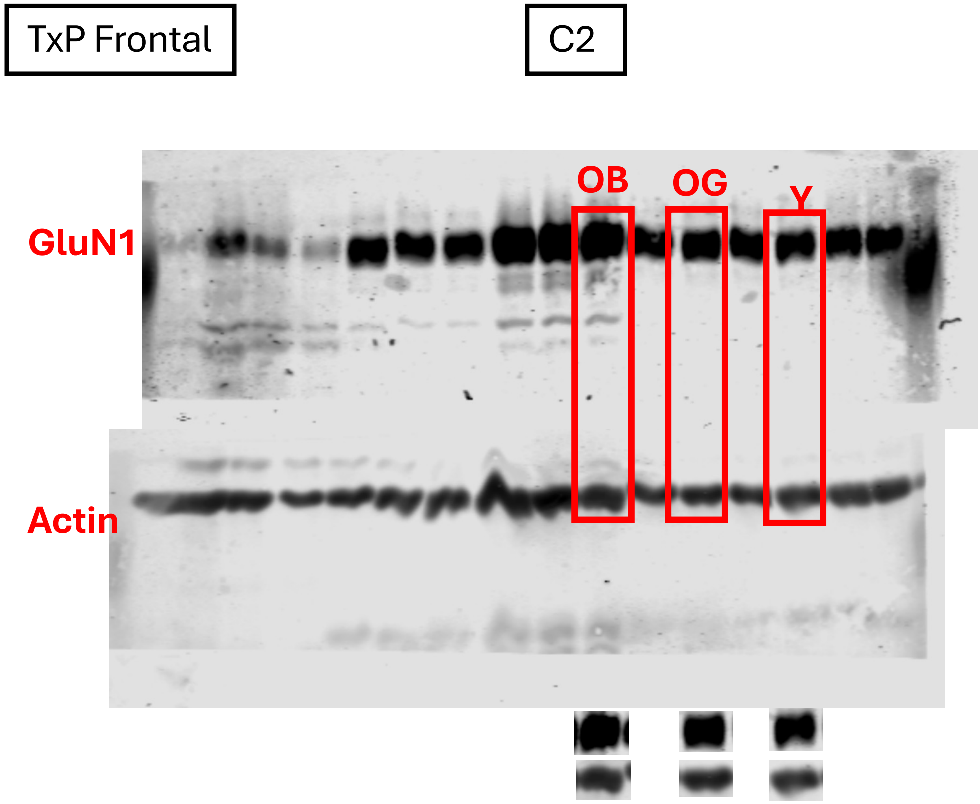
TxP Frontal
C2
OB
OG
Y
GluN1
Actin

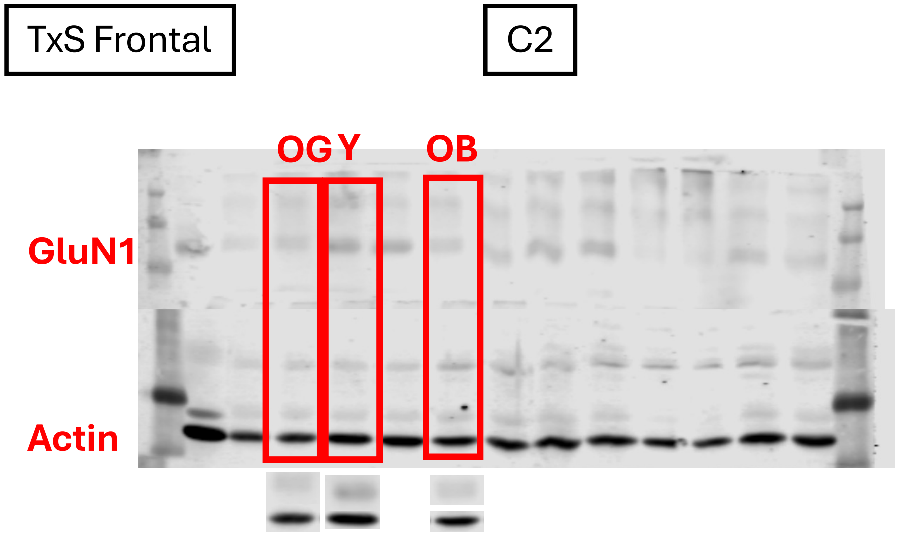
TxS Frontal
C2
OG
Y
OB
GluN1
Actin

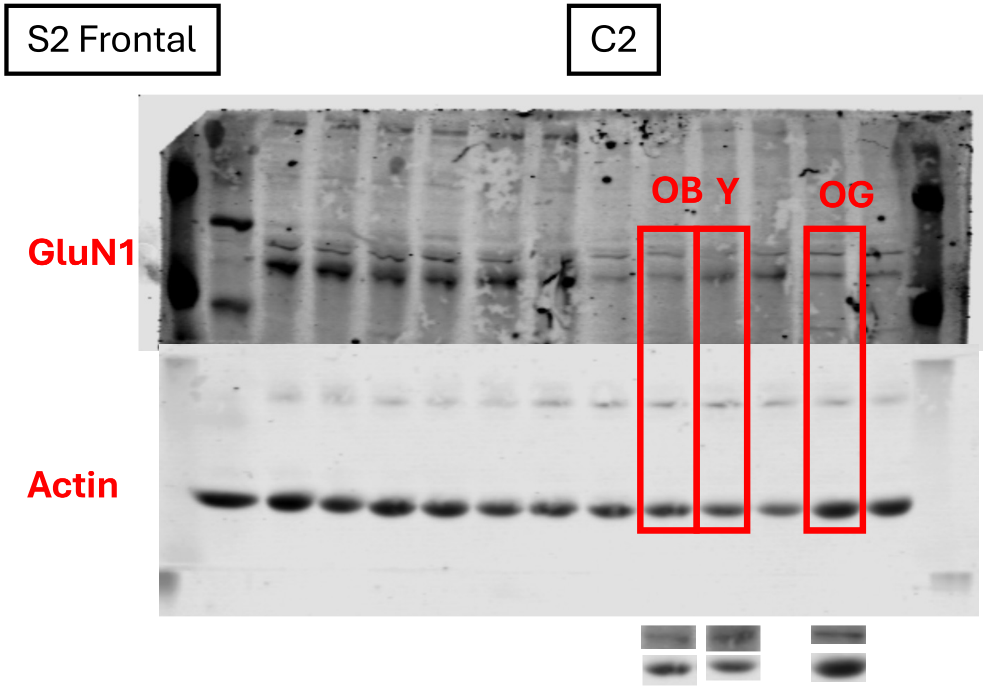
S2 Frontal
C2
GluN1
OB
Y
OG
Actin

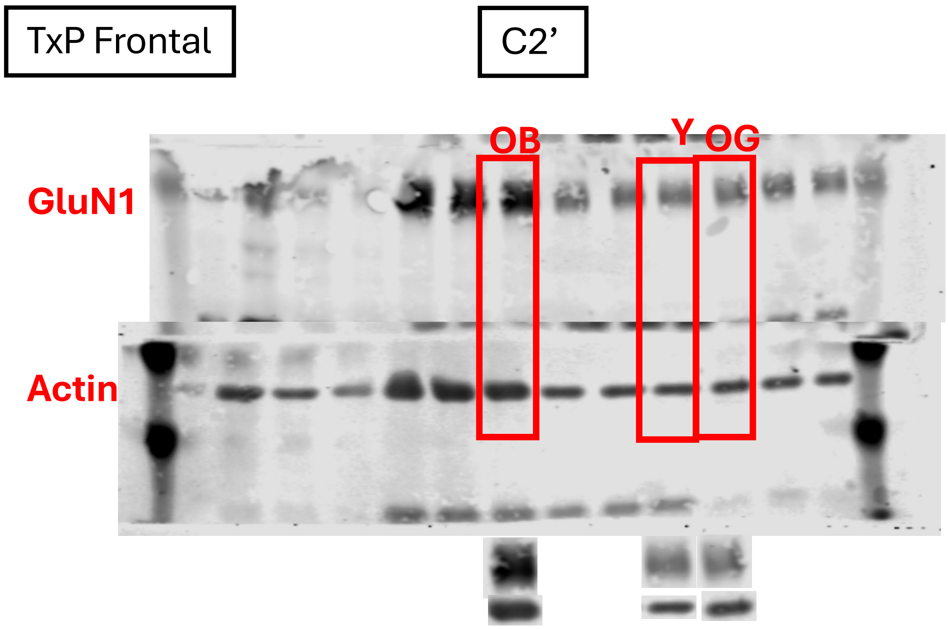

TxP Frontal
C2’
OB
Y
OG
GluN1
Actin

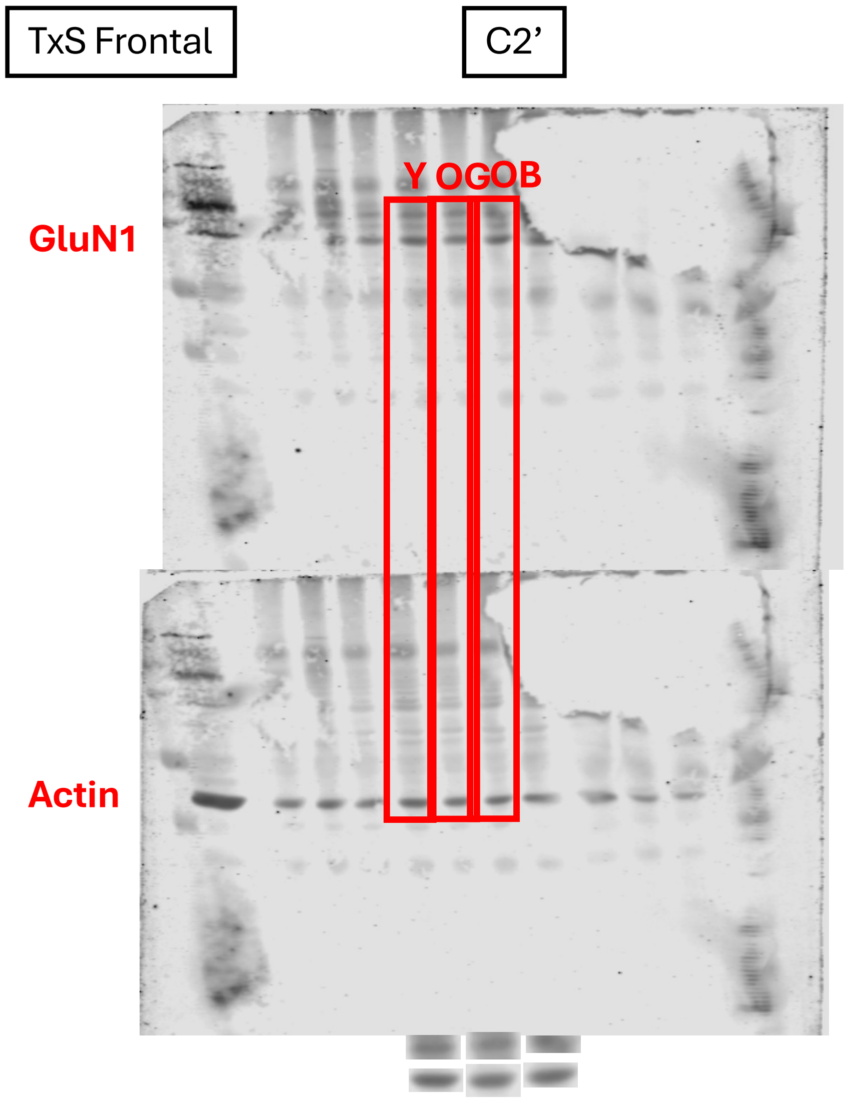
TxS Frontal
C2'
GluN1
Y
OG
OB
Actin

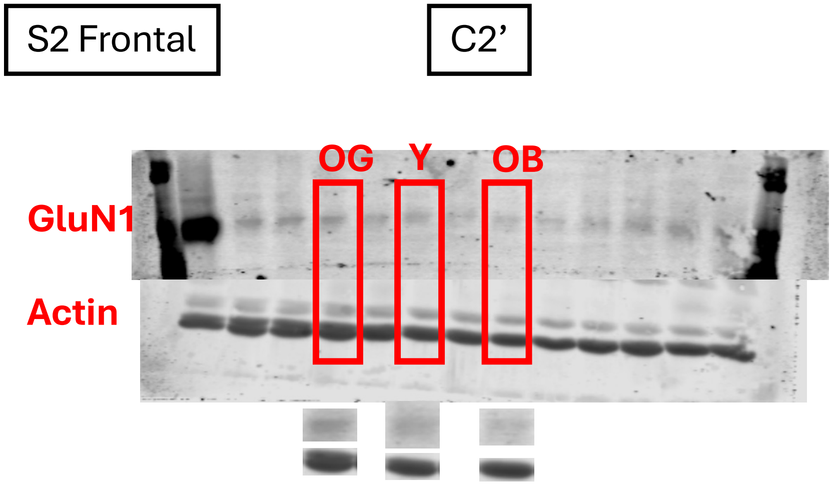
S2 Frontal
C2’
OG
Y
OB
GluN1
Actin

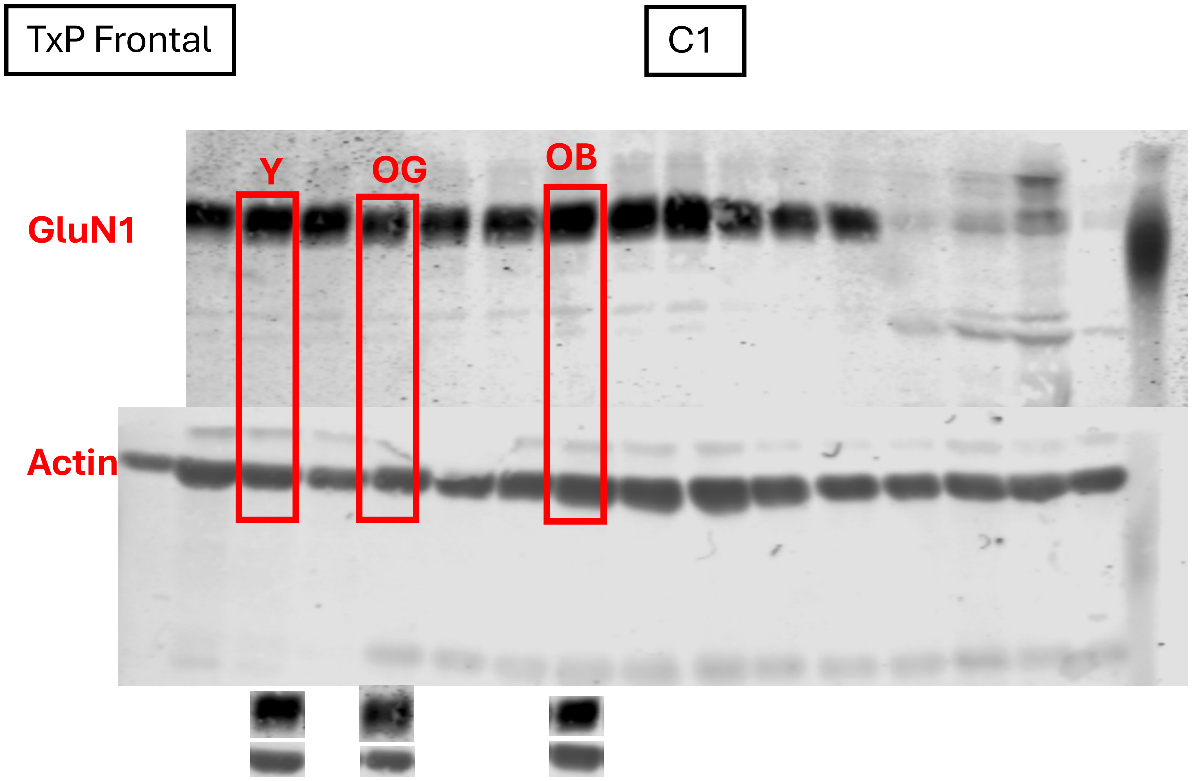
TxP Frontal
C1
GluN1
Actin
Y
OG
OB

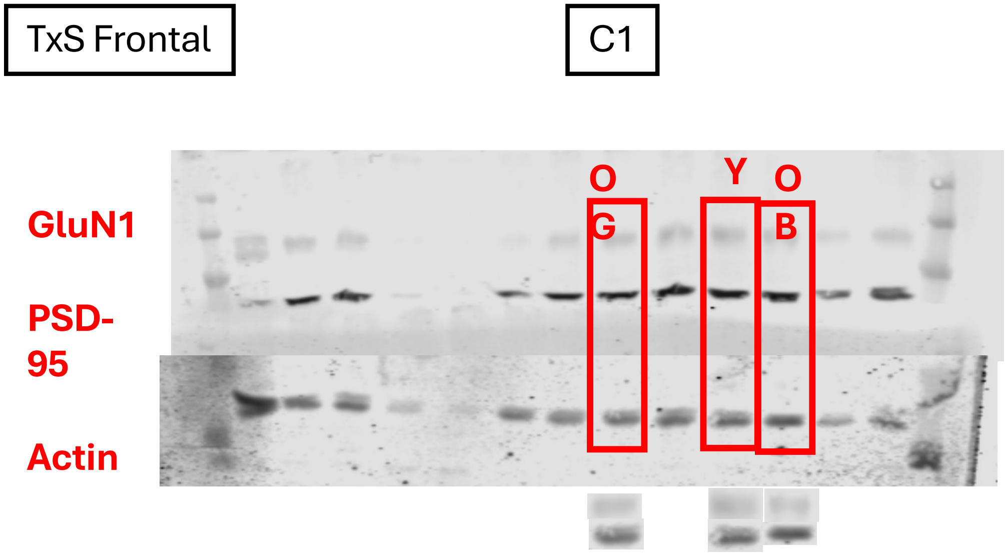
TxS Frontal
C1
GluN1
PSD-95
Actin
O
G
Y
O
B

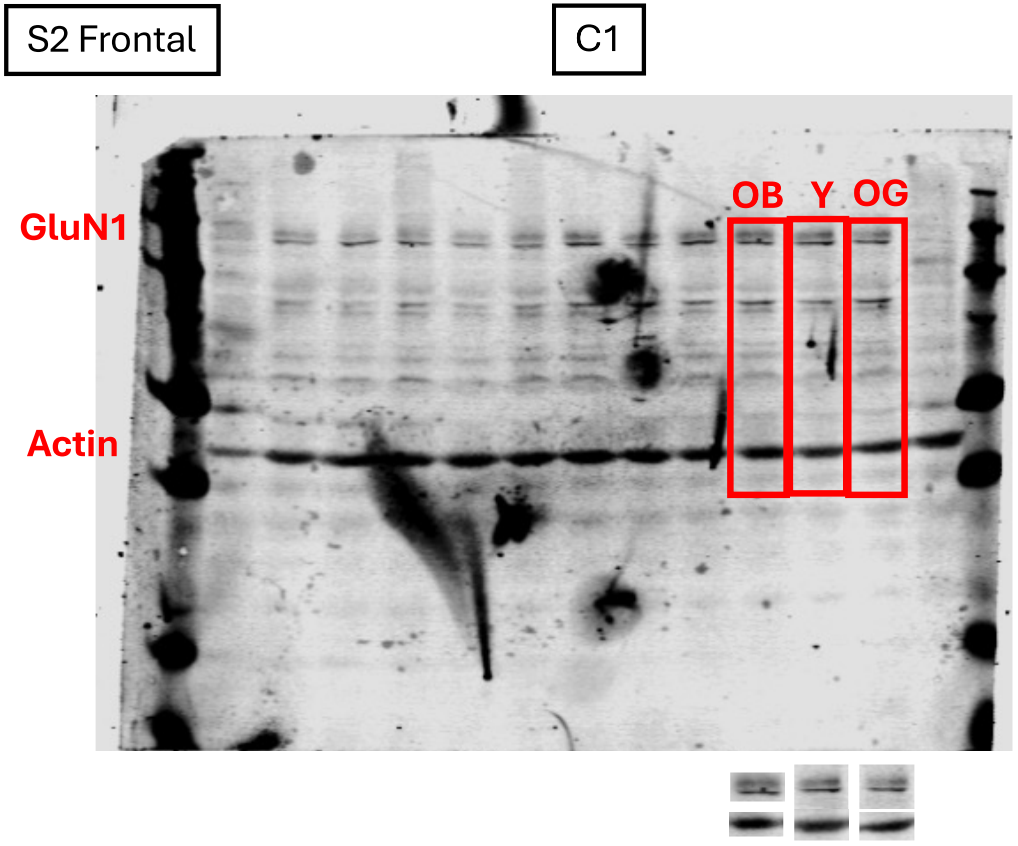
S2 Frontal
C1
GluN1
Actin
OB
Y
OG
